# Inflammation reprograms fibro-adipogenic progenitors to sustain immunopathogenic niches in myositis

**DOI:** 10.1038/s41419-026-08966-w

**Published:** 2026-06-12

**Authors:** Christopher Nelke, Julian Sanchez-Dal Cin, Charles-Antoine Dallevet, Jin-Soo Park, Jacqueline C. Kinold, Christina B. Schroeter, Felix Kleefeld, Anne-Katrin Güttsches, Paula Quint, Sara Walli, Derya Cengiz, Vera Dobelmann, Corinna Preusse, Alexander Mensch, Anne Schänzer, Markus Leo, Tim Hagenacker, Benedikt Schoser, Jörg H. W. Distler, Akiyoshi Uezumi, Werner Stenzel, Sven G. Meuth, Olivier Benveniste, Yves Allenbach, Tobias Ruck

**Affiliations:** 1 Ruhr University Bochum, BG University Hospital Bergmannsheil, Department of Neurology, Bochum, Germany; 2 BG University Hospital Bergmannsheil, Heimer Institute for Muscle Research, Bochum, Germany; 3 Department of Neurology, Medical Faculty, Heinrich Heine University Duesseldorf, Duesseldorf, Germany; 4 Sorbonne University, INSERM, Center of Research in Myology, UMRS 974, 75013 Paris, France; 5 Charité – Universitätsmedizin Berlin, corporate member of Freie Universität Berlin and Humboldt-Universität zu Berlin, Department of Neuropathology, Charitéplatz 1, 10117 Berlin, Germany; 6 Department of Neurology, University Medicine Halle, Halle (Saale), Germany; 7 Institute of Neuropathology, Justus-Liebig-University, Giessen, Gießen Germany; 8 Department of Neurology, University Medicine Essen, Center for Translational Neuro- and Behavioral Sciences (C-TNBS), Hufelandstr. 55, 45147 Essen, Germany; 9 Friedrich Baur Institute at the Department of Neurology, LMU University Hospital, LMU Munich, Munich, Germany; 10 Department for Rheumatology, University Hospital Düsseldorf, Medical Faculty of Heinrich Heine University, Düsseldorf, Germany; 11 Hiller Research Center, University Hospital Düsseldorf, Medical Faculty of Heinrich Heine University, Düsseldorf, Germany; 12 Division of Cell Heterogeneity, Medical Research Center for High Depth Omics, Medical Institute of Bioregulation, Kyushu University, 3-1-1 Maidashi, Higashi, Fukuoka, 812-8582 Japan

**Keywords:** Autoimmunity, Immunopathogenesis

## Abstract

Idiopathic inflammatory myopathies (IIMs) are autoimmune disorders defined by persistent muscle inflammation, fibrosis, and frequent resistance to current therapies. However, the mechanisms perpetuating disease activity despite immunosuppressive treatment remain elusive. Here, we describe a novel role for tissue-resident stromal cells, specifically fibro-adipogenic progenitors (FAPs), in sustaining skeletal muscle inflammation. Utilizing single-nucleus and spatial transcriptomics in 24 IIM patients and six non-diseased controls, we describe how FAPs adapt to their tissue context, favoring T-cell–centric programs in T-cell environments and myeloid programs in macrophage environments. At the spatial level, FAPs form inflammatory niches by co-localizing with muscle stem cells and activated macrophages, positioning them to participate in cell-to-cell communication with both immune and muscle cells. Trajectory and ligand-receptor analyses suggest a dual-input mechanism whereby infiltrating immune cells (via TGF-β) and myofibers (via epidermal growth factor (EGF)) converge on the AP-1 transcription factor to drive FAP differentiation toward a pro-inflammatory and pro-fibrotic phenotype. Mechanistically, exposure of primary human FAPs to TGF-β and EGF induces a primed state by altering the accessibility to AP-1 regulatory elements. Together, our findings reveal a previously unrecognized role of tissue-resident stromal cells in IIM, highlighting microenvironmental cross-talk centered on FAPs as a promising and actionable therapeutic target.

## Introduction

Idiopathic inflammatory myopathies (IIMs) encompass a heterogeneous group of autoimmune diseases characterized by immune-mediated muscle damage [1]. Despite therapeutic advances, a substantial proportion of patients experience chronic treatment-refractory disease [1, 2]. underscoring the need to explore alternative mechanisms that sustain pathology beyond canonical immune pathways.

While meaningful progress has been made in characterizing the immune landscape of IIMs, the role of tissue-resident cells remains underexplored. Fibro-adipogenic progenitors (FAPs) are the main population of mesenchymal stromal cells residing within skeletal muscle [3, 4]. In healthy muscle, FAPs support muscle stem cell (MuSC) function and define the tissue architecture by producing the extracellular matrix (ECM) [5, 6]. However, in pathological conditions, their dysregulation can trigger maladaptive remodeling and fibrosis, thereby amplifying muscle degeneration. Despite the emerging role of the fibroblast lineage in autoimmune diseases [7–9]. The contribution of FAPs to the pathogenesis of IIMs remains poorly characterized.

Recent studies in single-nucleus RNA sequencing (snRNA-seq) have enabled a more granular understanding of muscle-resident cell populations in IIMs [3, 10]. Building on these findings, we and others have demonstrated that in inclusion body myositis (IBM), the FAP niche expands and adopts a pathological ECM phenotype marked by altered collagen expression [3, 10]. The notion that stromal cells can perpetuate inflammation has also gained traction in other autoimmune conditions. In rheumatoid arthritis, for example, synovial fibroblasts assume a metabolically reprogrammed and epigenetically primed state, promoting inflammation and tissue destruction independently of immune cell activity [11]. This phenomenon, termed “tissue priming” [11, 12]. raises the possibility that a similar process may occur in skeletal muscle inflammation. Given the critical role of FAPs in maintaining muscle integrity and their capacity to adopt context-specific phenotypes, characterizing these phenotypes may provide key insights into the mechanisms driving chronic inflammation.

In this study, we employed snRNA-seq and spatial transcriptomics (ST) to profile FAP populations across three distinct IIM subtypes—anti-synthetase syndrome (ASYS), IBM, and immune-mediated necrotizing myopathy (IMNM)—and in non-diseased control (NDC) muscle tissue, aiming to define disease-specific FAP states. Our findings suggest that distinct inflammatory cues reprogram the FAP lineage towards maladaptive phenotypes, which may in turn amplify tissue inflammation across the IIM spectrum.

## Results

### The landscape of the myogenic and non-myogenic cells in the inflamed muscle

**Fig. 1:**
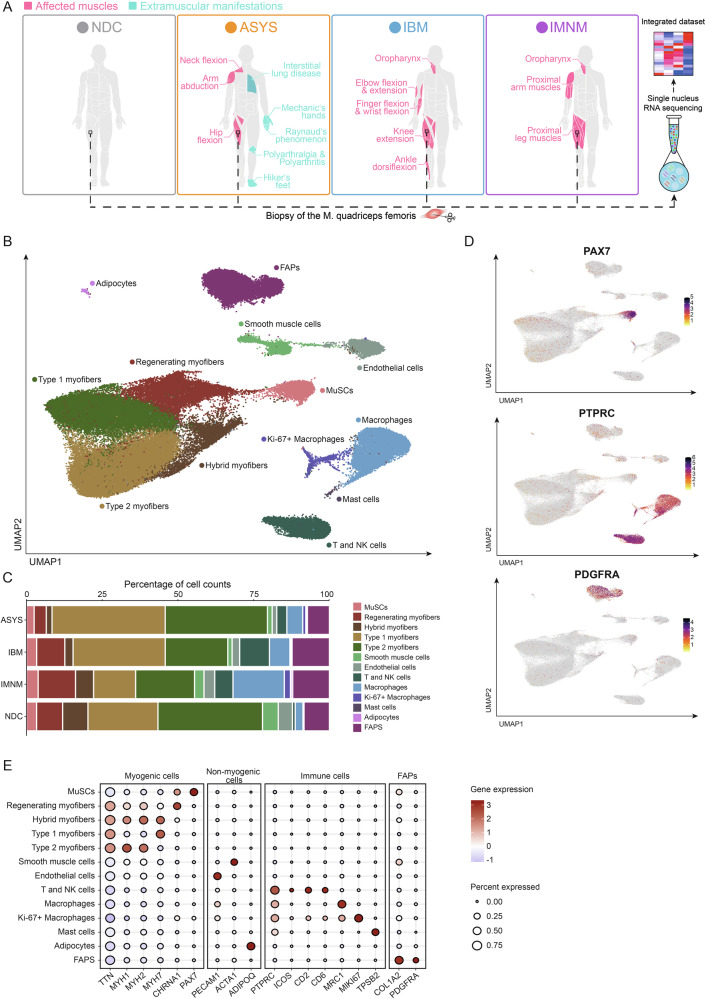
A transcriptomic atlas of inflamed muscle. **A** Overview of the study design and cohort. IIM patients with ASYS, IBM, and IMNM were included as diseased groups with NDCs as controls. The characteristic clinical features of each group are shown. **B** UMAP of the snRNA-seq atlas. Each dot is one cell, colored by its annotated cell type. Samples were integrated with Harmony. **C** Composition of each snRNA-seq dataset per group. The percentage of cell counts is shown as stacked bar plots. **D** Exemplary plots indicating the expression of marker genes. The normalized gene expression is shown. **E** Dot plot indicating the normalized expression of chosen marker genes and the percentage of cells per cell type expressing these genes. ASYS anti-synthetase syndrome, IBM inclusion body myositis, IIM idiopathic inflammatory myopathies, IMNM immune-mediated necrotizing myopathy, NDC non-diseased control, snRNA-seq single nuclei RNA sequencing, UMAP uniform manifold approximation and projection.

Our aim was to profile FAPs across the IIM spectrum and investigate their context-specific phenotypes. To capture the spectrum of disease heterogeneity, we selected three clinically and immunologically distinct IIM subtypes: ASYS (*n* = 5), IMNM (*n* = 11), and IBM (*n* = 8). ASYS represents an acute-to-subacute IIM with systemic features; IMNM is characterized by primary muscle involvement and variable clinical severity; and IBM is distinguished by its treatment-refractory, T-cell–driven pathology [2]. Detailed characteristics of our cohort are provided in Table 1 and align with prior knowledge on clinical presentations (Fig. 1A).

**Table 1:** Clinical and demographic characteristics.

	ASYS	IBM	IMNM	NDC
Number of patients	5	8	11	6
Number of samples analysed by snRNA-seq	3	6	9	4
Number of samples analysed by spatial RNA-seq	2	2	2	2
Median age at diagnosis (years, range)	48 (31 – 76)	71 (45 – 87)	65 (33 – 85)	68 (44 – 86)
Gender, *n* (%)				
Female	3 (60%)	4 (50%)	6 (55%)	3 (50%)
Male	2 (40%)	4 (50%)	5 (45%)	3 (50%)
Creatine kinase				
(U/I, mean, SD)*	402 (103)	218 (98)	497 (205)	87 (52)
Antibody status, *n* (%)				
Anti-cN-1A	0	4 (50%)	0	0
Anti-SRP	0	0	6 (55%)	0
Anti-HMGCR	0	0	2 (18%)	0
Anti-Jo1	5 (100%)	0	0	0
Seronegative	0	4 (50%)	3 (27%)	6 (100%)
Treatment, *n* (%)*				
Treatment naïve	0	6 (75%)	0	6 (100%)
IVIG	2 (40%)	2 (25%)	7 (63%)	0
Azathioprine	1 (20%)	0	0	0
Steroids	3 (60%)	0	4 (37%)	0
Steroid dose per day in mg (mean, SD)	10 (0)	0	8 (8)	0

*At time of biopsy abbreviations: *ASYS* anti-synthetase syndrome, *HMGCR* HMG-CoA reductase, IBM inclusion body myositis, *IMNM* immune-mediated necrotizing myopathy, *NDC* non-diseased control, *SD* standard deviation, *SRP* signal recognition particle.

We performed snRNA-seq on muscle biopsies, generating an atlas of approximately 96,000 nuclei from both IIM and control samples. This integrated atlas encompasses the cellular environment of skeletal muscle, encompassing myogenic populations—including myofiber subtypes and muscle stem cells (MuSCs)—as well as non-myogenic populations such as immune, vascular, and tissue-resident cells, including FAPs (Fig. 1B). Clustering and cell type annotation were guided by expression of canonical marker genes, consistent with prior transcriptomic studies on muscle tissue [3, 10, 13]. Exemplary marker genes are shown for MuSCs (*PAX7*), leukocytes (*PTPRC* encoding CD45), and FAPs (*PDGFRA*), supporting the classification approach (Fig. 1C–E).

We also examined the cell type composition across conditions to identify disease-associated shifts in the muscle environment (Fig. 1C). All IIM subtypes showed a marked expansion of immune cells relative to NDCs. In ASYS and IBM, this was driven predominantly by T and NK cells. In contrast, IMNM displayed a prominent expansion of macrophages, in line with its histopathological profile [14]. Notably, we identified a distinct subset of Ki-67⁺ macrophages almost exclusively in IMNM, suggesting an activated state for this immune cell type. In the myogenic compartment, IMNM displayed a high number of regenerating myofibers, validating observations from histopathological studies [14, 15]. Additionally, our data reaffirm previous findings of fiber-type alterations in IBM, where a reduction in type 2 myofibers accompanies a relative predominance of type 1 fibers, as reported by us and others [3, 10]. In summary, our snRNA-seq atlas recapitulates known hallmarks of IIM pathology and provides a granular platform for dissecting disease-specific alterations in muscle-resident cell states. The complete dataset is publicly available at https://tinyurl.com/FAPAtlas.

### FAPs engage distinct phenotypes across the IIM spectrum

**Fig. 2:**
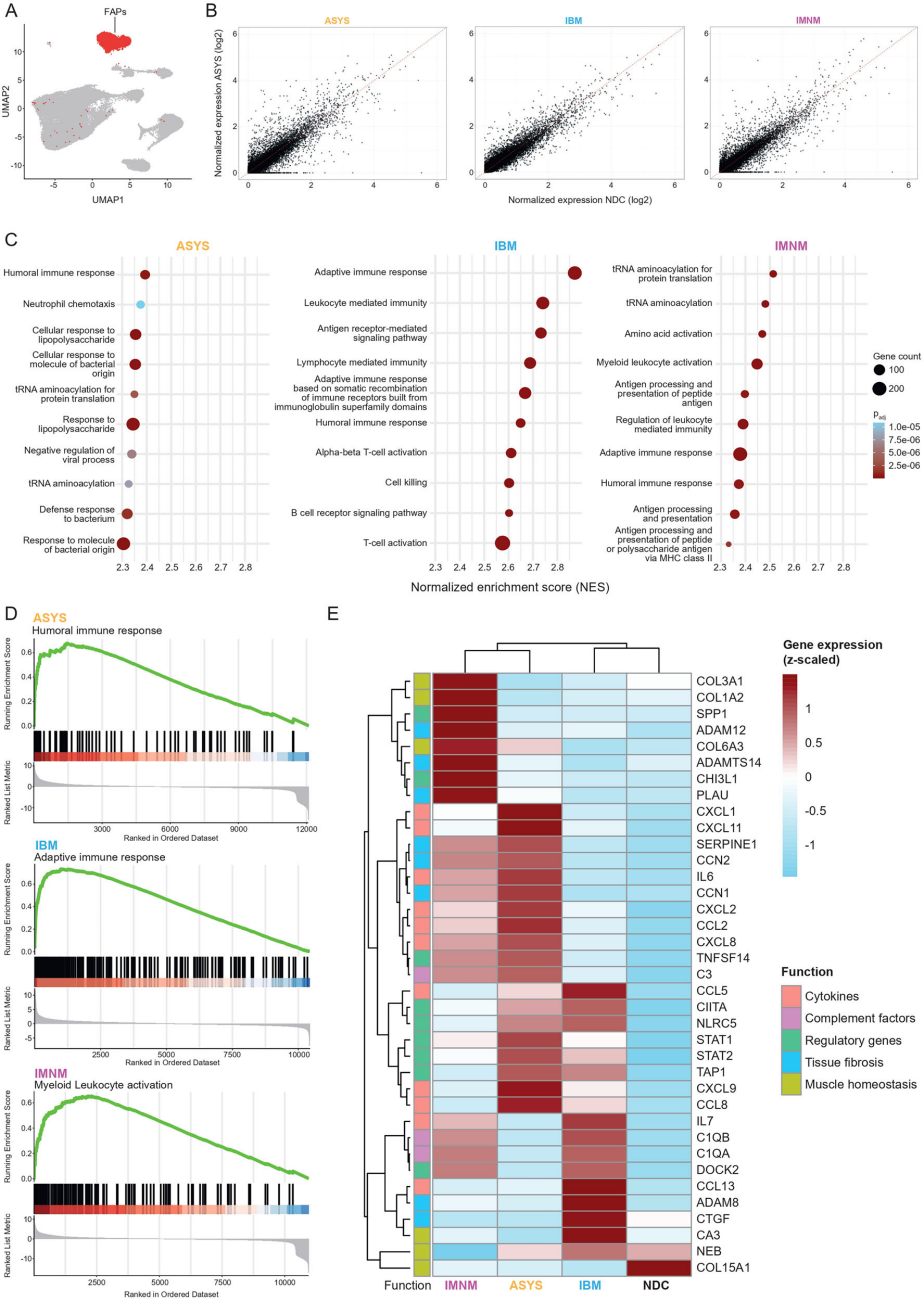
FAPs acquire distinct phenotypes in IIMs. **A** FAPs were extracted from the full dataset for analysis. **B** Scatter plots indicating the fold change of DEGs comparing NDC to ASYS, IBM and IMNM, respectively. The normalized gene expression for NDC is displayed on the X-axis, and the normalized gene expression for the respective disease group on the Y-axis. **C** GSEA analysis of the significant DEGs for each IIM subtype for the GO BP database. The top ten pathways sorted by the NES are shown. The NES normalizes the raw Enrichment Score to account for differences in gene set size and correlations between gene sets. **D** Running score and rank shown for selected GO BP pathways. The IIM subtype indicated above each plot, compared to NDC, is shown for the respective GO BP pathway. **E** Heatmap showing the genes with the highest variance in the FAP cell population. The normalized gene expression is indicated by color code. Each gene was annotated by its function. ASYS anti-synthetase syndrome, DEGs differentially expressed genes, GO BP Gene Ontology Biological Processes, IBM inclusion body myositis, IMNM immune-mediated necrotizing myopathy, NDC non-diseased control, NES normalized enrichment score.

To investigate the heterogeneity of FAPs across the IIM spectrum, we isolated this population from our snRNA-seq dataset and characterized their transcriptomic signatures (Fig. 2A). By calculating differentially expressed genes (DEGs) for each IIM subtype relative to NDCs, we identified distinct transcriptional programs associated with each condition (Fig. 2B).

To contextualize these differences, we performed GSEA, assigning DEGs to biological pathways (Fig. 2C and Suppl. File 1). FAPs from ASYS patients were enriched for pathways linked to the humoral immune response, neutrophil chemotaxis, and tRNA aminoacylation. In IBM, FAPs exhibited a transcriptomic reprogramming towards the adaptive immune response, leukocyte-mediated immunity, and T cell activation. In IMNM, FAPs shifted towards the activation of myeloid leukocyte pathways, alongside the adaptive immune response. Collectively, these findings suggest that FAPs adapt their phenotype to their disease-specific immune environments: IBM FAPs corresponded to a T cell–driven pathology, IMNM FAPs aligned with macrophage-driven inflammation, and ASYS FAPs paralleled a humoral, B cell–associated pathology. Surprisingly, a shared feature between ASYS and IMNM was the enrichment of pathways involved in tRNA aminoacylation. This conserved process, catalyzed by aminoacyl-tRNA synthetases (ARSs), ensures accurate protein synthesis by linking amino acids to their corresponding tRNAs [16]. ARSs are essential enzymes that catalyze the binding of specific amino acids to tRNAs. Beyond this canonical function, ARSs have been increasingly implicated in immune modulation and the regulation of immune cell development [16].

Further, we previously reported that FAPs enter cellular senescence in IBM [3]. To validate this observation in this cohort, we assessed the enrichment of a curated senescence gene signature (SenMayo) across disease subtypes, using NDCs as a baseline. Consistent with prior findings, FAPs in IBM were enriched for senescence-associated biomarkers, whereas no such enrichment was observed in ASYS or IMNM, suggesting that a subset of FAPs undergoes senescence specifically in IBM (Suppl. Fig. 1).

We next examined the most significantly upregulated genes in FAPs across each IIM subtype (Fig. 2E). Each FAP phenotype exhibited a specific regulatory pattern favoring immune- and fibrosis-related genes. For instance, ASYS FAPs upregulated *IL6*, a pro-inflammatory cytokine previously reported at elevated levels in ASYS muscle biopsies and known to stimulate lymphocyte activation [17]. IBM FAPs expressed chemokines such as *CCL13*, a T cell chemoattractant, and *IL7*, a cytokine driving lymphoid cell proliferation. In ASYS and IMNM, FAPs also engaged *CCL2* expression, a marker for a pro-inflammatory FAP phenotype [18]. Moreover, FAPs across all IIM subtypes consistently downregulated *COL15A1* compared to NDCs, aligning with prior observations in IBM muscle [3, 10].

Together, these data support that FAPs adopt distinct, disease-specific phenotypes across IIMs, mirroring their inflammatory context and potentially acting as local amplifiers of immunopathology.

### Inflammatory cues drive distinct cell fates of the FAP lineage

**Fig. 3:**
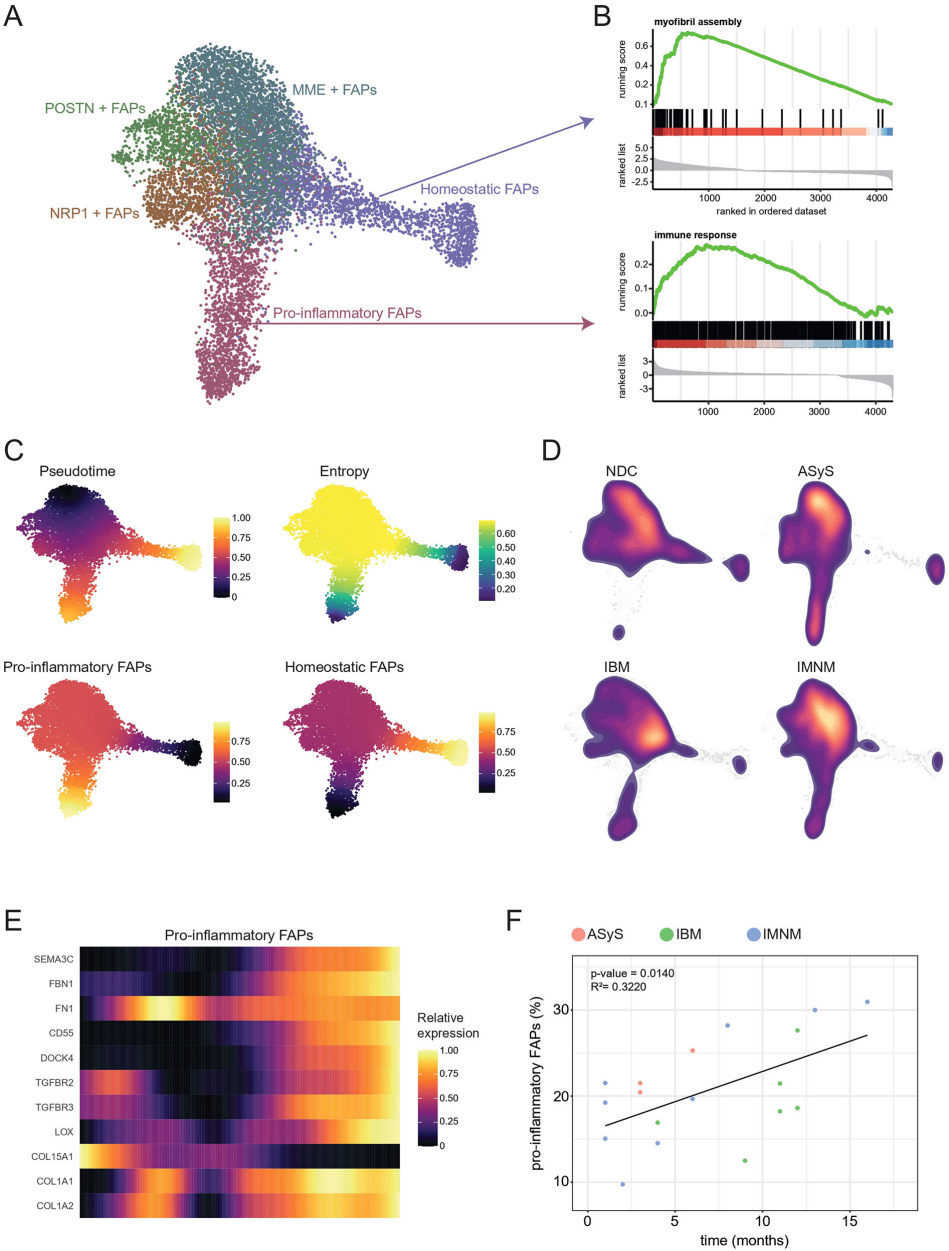
Diverging trajectories of the FAP lineage. **A** UMAP of the FAP population. All FAPs were extracted from the full dataset and re-analysed. The full population was subclustered. **B** GSEA analysis of the indicated GO BP pathways showing the running score in the ranked dataset. The upper GSEA shows the comparison of homeostatic FAPs to all other FAPs, while the lower plot shows the comparison of pro-inflammatory FAPs to all others. **C** UMAP indicating the pseudotime and entropy scores and commitment to the pro-inflammatory and homeostatic cell fates. The pseudotime is indicated as a normalized score ranging from 0 to 1, with lower values indicating early cells along the trajectory. The lower panels show the lineage-specific pseudotime. **D** Density plots showing the cell density for each IIM subtype and NDCs. **E** Relative gene expression along the pseudotime of the pro-inflammatory trajectory of the indicated genes. Gene expression was scaled from 0 to 1. **F** Univariate linear regression for the percentage of pro-inflammatory FAPs and the disease duration, defined as the time between the reporting of first symptoms and the time of muscle biopsy. Significance was tested by the likelihood test. *R*^2^ is indicated in the plot. ASYS anti-synthetase syndrome, IBM inclusion body myositis, IMNM immune-mediated necrotizing myopathy, NDC non-diseased control.

Building on our observation that FAPs exhibit disease-specific transcriptomic phenotypes, we next sought to understand how inflammatory cues shape the developmental trajectories of this lineage. To this end, we performed subclustering and trajectory analysis focused on the FAP population, aiming to resolve cellular heterogeneity and identify state transitions influenced by inflammation (Fig. 3A). Some genes and pathways identified in the previous analysis reappear in the trajectory-based framework. This convergence is expected, as inflammation- and fibrosis-associated genes not only distinguish disease-specific FAP subsets but also define endpoints along their fate trajectories.

This dimensionality reduction revealed both previously recognized FAP states and novel subpopulations that emerged under inflammatory conditions. Notably, there is currently no consensus on the transcriptomic taxonomy of the FAP lineage, with various studies applying different nomenclatures and gene markers [3, 10, 13, 19]. For consistency, we annotated the main FAP population as *MME*^*+*^ (membrane metalloendopeptidase), a previously described subset [4]. and identified two closely related subpopulations defined by the expression of *POSTN* (periostin) and *NRP1* (neuropilin 1), respectively (Suppl Fig. 2A).

We observed two distinct developmental trajectories emerging from the main FAP population. In one trajectory, cells differentiated toward a homeostatic state. This FAP cluster was characterized by *DPP4*, a marker of resting FAP populations [20]. These FAPs exhibited strong enrichment for gene programs associated with myofibril assembly, muscle cell development, and sarcomere organization (Suppl. Fig. 2B), suggesting a supportive role in myofiber differentiation. In contrast, the second trajectory downregulated genes related to muscle structural development and instead engaged a transcriptional program characteristic of an activated, pro-inflammatory state. This was evidenced by enrichment for pathways involved in cell activation, the immune response, and regulation of inflammatory signaling. The pro-inflammatory FAP cluster was defined by expression of *CXCL1*, *CCL2*, *IL18,* or *LIF*, among others.

To further understand how these fates emerge, we performed pseudotime and fate mapping analysis (Fig. 3C). Here, one terminal branch aligned with the activated, pro-inflammatory state, while the other aligned with the homeostatic FAP state. Divergence into these fates was accompanied by a progressive reduction in entropy, suggesting increasing transcriptional commitment and mutual exclusivity between trajectories. Notably, the distribution of FAP states varied across disease subtypes (Fig. 3D). Across IIMs, we observed a similar expansion of pro-inflammatory FAPs, while NDCs retained a higher proportion of homeostatic FAPs. As reflected by the cell density, FAP differentiation was most pronounced in IBM, with the majority of cells having committed to either the homeostatic or pro-inflammatory fate. Taken together, the inflammatory muscle milieu appears to guide FAPs toward distinct fate commitments, with terminal differentiation particularly prominent in IBM.

To dissect the transcriptional programs driving these trajectories, we examined gene expression dynamics along each path (Fig. 3E and Supplementary Fig. 2C). The pro-inflammatory trajectory was marked by progressive upregulation of fibrosis-associated genes, such as fibronectin (*FN1*), collagen type I isoforms (*COL1A1* and *COL1A2*), and lysyl oxidase (*LOX*). The latter facilitates cross-linking of extracellular-matrix proteins such as collagen, and its dysregulation leads to excessive matrix stiffening and fibrotic remodeling [21, 22]. Concurrently, this trajectory was characterized by a loss of collagen type XV (*COL15A1*). In addition to fibrotic remodeling, pro-inflammatory FAPs upregulated genes such as dedicator of cytokinesis protein 4 (*DOCK4*), known to regulate cell adhesion [23]. or semaphorin3C (*SEMA3C*), promoting immune cell differentiation [24, 25]. These changes occurred in a fate-dependent manner and were absent in the homeostatic branch. Notably, the pro-inflammatory FAPs also exhibited upregulation of the TGF-β receptors (*TGFBR2* and *TGFB3*), suggesting that TGF-β signaling may contribute to the induction or stabilization of this cell fate. Supporting this notion, expression of fibrillin-1 (*FBN1*), a matrix-associated protein involved in extracellular matrix remodeling and known to be regulated by TGF-β [26, 27], was also progressively upregulated along the pro-inflammatory trajectory.

Finally, to explore how these cell fates are linked to clinical features, we tested the hypothesis that persistent inflammation drives the accumulation of pro-inflammatory FAPs. Indeed, we observed a positive correlation between the disease duration (defined as the time between first reporting of symptoms and the time of the muscle biopsy) and the proportion of pro-inflammatory FAPs across IIM subtypes (Fig. 3F). In contrast, patient age did not correlate with the proportion of pro-inflammatory FAPs (*p* = 0.84, *R*^2^ = 0.002).

Collectively, these data suggest that persistent inflammatory cues drive FAPs toward a transcriptionally committed, pro-inflammatory and pro-fibrotic cell fate. This commitment is particularly evident in IBM.

### Cell-to-cell communication implicates TGF-β and EGF in FAP differentiation

Next, we sought to investigate the extrinsic factors shaping these fate decisions. Given that immune cell infiltration is a defining feature of the IIM spectrum [1, 2]. We hypothesized that these cells constitute the principal source of differentiation cues within the inflamed muscle microenvironment.

To explore this, we performed cell-to-cell communication (CCC) analysis using NicheNet [28]. a computational framework that integrates known ligand-receptor interactions with downstream gene regulatory networks to predict how signals from one cell population influence the transcriptional programs of another. In this model, we positioned immune cells (lymphocytes and macrophages) as the sender population and FAPs as receivers. We applied this framework separately to each disease subtype—ASYS, IBM and IMNM—with NDC as baseline reference.

**Fig. 4:**
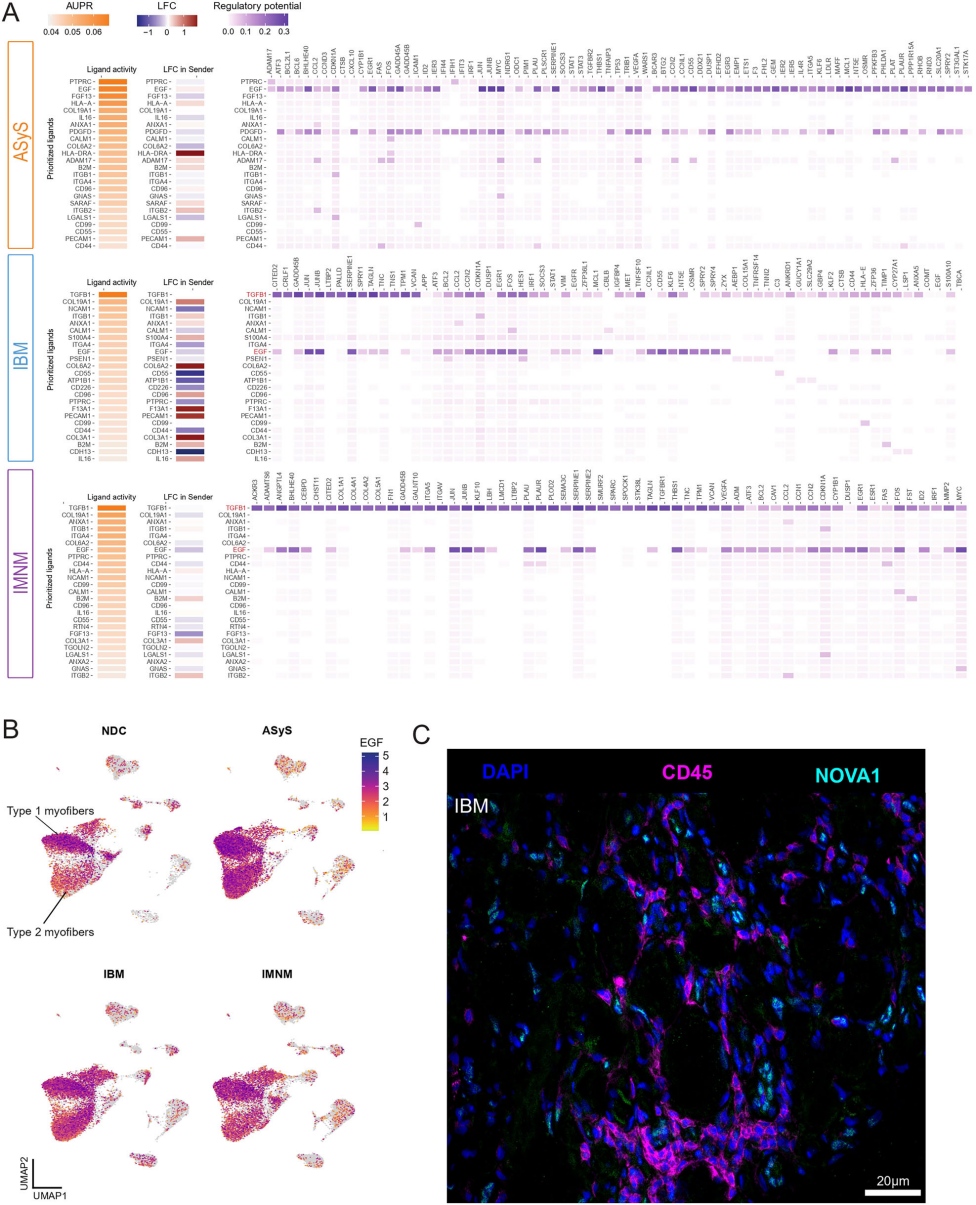
Cell-to-cell communication favors TGF-β and EGF signaling. **A** NichNet analysis of the cell-to-cell communication. Lymphocytes were set as the sender cell type and FAPs as the receiver cell type. Analysis was performed for each IIM subtype, as indicated, compared to NDC. Results are shown for the 20 ligands best predicting the FAP transcriptome, ranked by the Pearson correlation coefficient, indicating the ability of each ligand to predict target genes. The second column indicated the log fold change (LFC) for each gene in the lymphocyte cell cluster comparing each IIM subtype to NDC as indicated. The ligand–target matrix indicates the regulatory potential between ligands and target genes from the FAP transcriptome. **B** Feature plot indicating the expression of EGF in each group. The normalized gene expression is shown. **C** Representative immunofluorescence image of an IBM muscle biopsy. The target proteins are indicated. The scale bar shows 20 µm. ASYS anti-synthetase syndrome, FAP fibro-adipogenic progenitor, IBM inclusion body myositis, IMNM immune-mediated necrotizing myopathy, NDC non-diseased control, NOVA1 RNA-binding protein Nova1, EGF epidermal growth factor, TGF transforming growth factor.

In our CCC model (Fig. 4A), we identified two major signaling pathways predicted to drive the transcriptional reprogramming of the FAP lineage: TGF-β and epidermal growth factor (EGF). In both IBM and IMNM, TGF-β ligands demonstrated the highest predicted activity, with downstream induction of gene programs associated with inflammation and fibrosis. Specifically, TGF-β signaling was predicted to regulate target genes involved in FAP activation (e.g., *CCL2*) and the pro-inflammatory phenotype (e.g., *SEMA3C*), as well as extracellular matrix remodeling, including a shift toward type I collagen (e.g., *COL1A1* and *COL1A2*). In addition, TGF-β appeared to induce a cellular stress response converging on target genes of the AP-1 transcription factor (TF), evidenced by upregulation of *JUN/JUNB, FOS/FOSB*, as well as *GADD45A/GADD45B*, and *CDKN1A* (p21). GADD45A and p21 are also implicated in establishing cellular senescence [29]. Together, these findings suggest that TGF-β is a principal ligand driving the emergence of pathogenic FAP states. While the strongest predicted ligand-receptor activity arose from TGF-β, this signaling appeared to be amplified by EGF, although with lower inferred ligand activity from immune cells. This is consistent with the known tissue distribution of EGF, which is also secreted by myofibers in skeletal muscle [30, 31]. Therefore, the presence of EGF-regulated target genes in FAPs likely reflects signaling cues from the myogenic compartment that converge in the observed FAP responses. Indeed, we detected EGF expression primarily in myofibers (Fig. 4B). In NDC, type 1 myofibers express EGF, while only low expression is detected in type 2 myofibers. In contrast, EGF is consistently detected in both myofiber types in IIMs, suggesting that type 2 myofibers contribute to EGF signaling in inflamed muscle.

In contrast to the CCC models observed in IBM and IMNM, ASYS presented a more heterogeneous signaling landscape. Here, multiple ligands, including CD45 (PTPRC), EGF, platelet-derived growth factor D (PDGFD), and tumor necrosis factor (TNFSF10), were predicted to converge on a similar set of target genes. These receptor-ligand interactions were also predicted to induce genes favoring cell activation and ECM remodeling.

We validated the spatial co-localization of immune cells and FAPs by immunofluorescence (IF). Leukocytes were identified by CD45, while we chose RNA-binding protein Nova1 (NOVA1) as a marker for the FAP lineage. NOVA1 emerged as the most significant FAP lineage marker in our dataset, motivating this choice (Supplementary Fig. 3A, B). NOVA1^+^ FAPs were abundant within inflammatory infiltrates (Fig. 4C), whereas they were scarce in NDC tissue (Supplementary Fig. 3C, D). Notably, NOVA1^+^ FAPs tended to cluster in proximity to CD45+ aggregates, supporting a spatially organized crosstalk between FAPs and immune cells.

Taken together, these results suggest that distinct ligands shape the emergence of disease-specific FAP phenotypes. These findings position FAP reprogramming as a downstream consequence of immune cell infiltration and inflammatory signaling in IIMs.

### Validation and spatial co-localization of FAPs

**Fig. 5:**
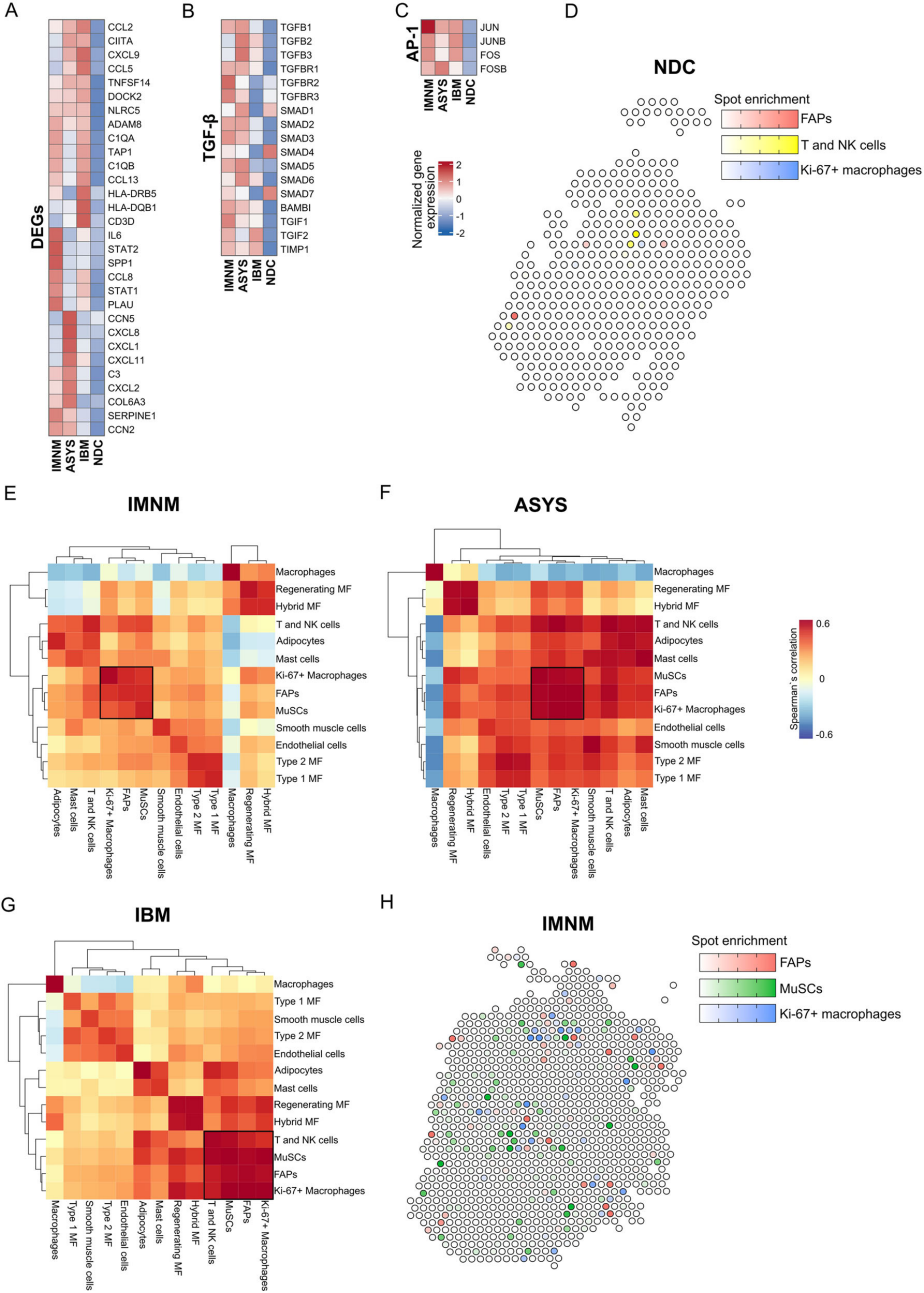
The spatial transcriptome of FAPs in IIMs. **A** Heatmap of normalized gene expression comparing FAP spots in the spatial transcriptomics datasets for the indicated genes. All spots enriched for FAPs were analysed per group. **B** Heatmap of normalized gene expression for genes related to TGF-β signaling. **C** Heatmap of normalized gene expression for genes related to AP-1 activity. *N* = 2 per group. **D** Exemplary overlay for NDC showing enrichment for the indicated cell types per spot. **E**–**G** Heatmaps showing Spearman’s correlation for each IIM subtype. Correlation was correlated between the indicated cell types. A black box highlights the correlation of FAPs with surrounding cells. **H** Exemplary overlay for IMNM showing enrichment for the indicated cell types per spot. *N* = 2 per group. AP-1 activator protein 1, ASYS anti-synthetase syndrome, FAP fibro-adipogenic progenitor, IBM inclusion body myositis, IMNM immune-mediated necrotizing myopathy, NDC non-diseased control, TGF-β transforming growth factor beta.

To cross-validate our results and place them into a spatial context, we profiled muscle biopsies from each group using ST (*n* = 2 per group). We annotated spatial spots with the same marker-gene strategy as in our snRNA-seq dataset to enable harmonization across modalities. Based on our snRNA-seq data, we performed differential expression testing and found that FAP-enriched spots in IIM recapitulate a pro-inflammatory and pro-fibrotic profile. Specifically, IIM samples showed an altered collagen signature with *COL15A1* downregulated versus NDC, and increased expression of genes required for antigen-presentation via MHC class I (*NLRC5* and *TAP1*) and MHC class II (*CIITA*), with the latter detected in IBM and ASYS but not in IMNM, consistent with previous studies [32]. Further, FAPs in IIM displayed a distinct cytokine repertoire relative to NDC, including members of the CXCL and CCL chemokine families as well as IL6 and IL7 (Fig. 5A and Suppl. File 2).

Building on our prior data suggesting that AP-1 regulation and TGF-β signaling may shape FAP phenotypes, we also analysed these pathways. Our ST data showed higher expression of TGFB ligands, TGFBR receptors, and downstream TGF-β mediators compared with NDC (Fig. 5B). Consistently, AP-1 components (*JUN*, *JUNB*, *FOS*, and *FOSB*) were elevated in IIM FAPs across IIMs relative to NDC (Fig. 5C).

To understand how FAPs are embedded within the inflammatory microenvironment in IIMs, we performed a neighborhood analysis of our ST data. In NDCs, T and NK cells as well as activated macrophages were largely absent or confined to blood vessels (Fig. 5D and Supplementary Fig. 4), and FAPs were sparse compared to IIM samples. Across all three IIM subtypes, FAP expanded and co-localized with MuSCs and Ki-67⁺ macrophages (Fig. 5E–H), consistent with a pro-fibrotic loop in which FAPs attract immune cells via CCL- and CXCL-family chemokines and, reciprocally, receive pro-fibrotic signals such as TGF-β from these neighboring immune cells. Notably, in IBM, but not in ASYS or IMNM, FAPs also co-localized with T and NK cells, consistent with the T cell predominant pathophysiology of IBM. At the same time, we observed that macrophages co-localize with regenerating and hybrid MF in our ST dataset, consistent with known histopathological observations [32].

Taken together, ST profiling of FAPs recapitulates a pro-inflammatory FAP phenotype and places these FAPs in a spatial microenvironment with immune cells and MuSCs, supporting a specialized cellular niche.

### TGF-β and EGF enable access to the AP-1 transcription factor in vitro

Guided by our CCC model, we hypothesized that the AP-1 TF may orchestrate the response of FAPs in IIMs. AP-1 comprises c-Jun and c-Fos family proteins and related bZIP (basic leucine zipper domain) partners that heterodimerize and integrate signals from cytokines and stress. AP-1 also controls a fibrotic program in fibroblasts [33–36].

**Fig. 6:**
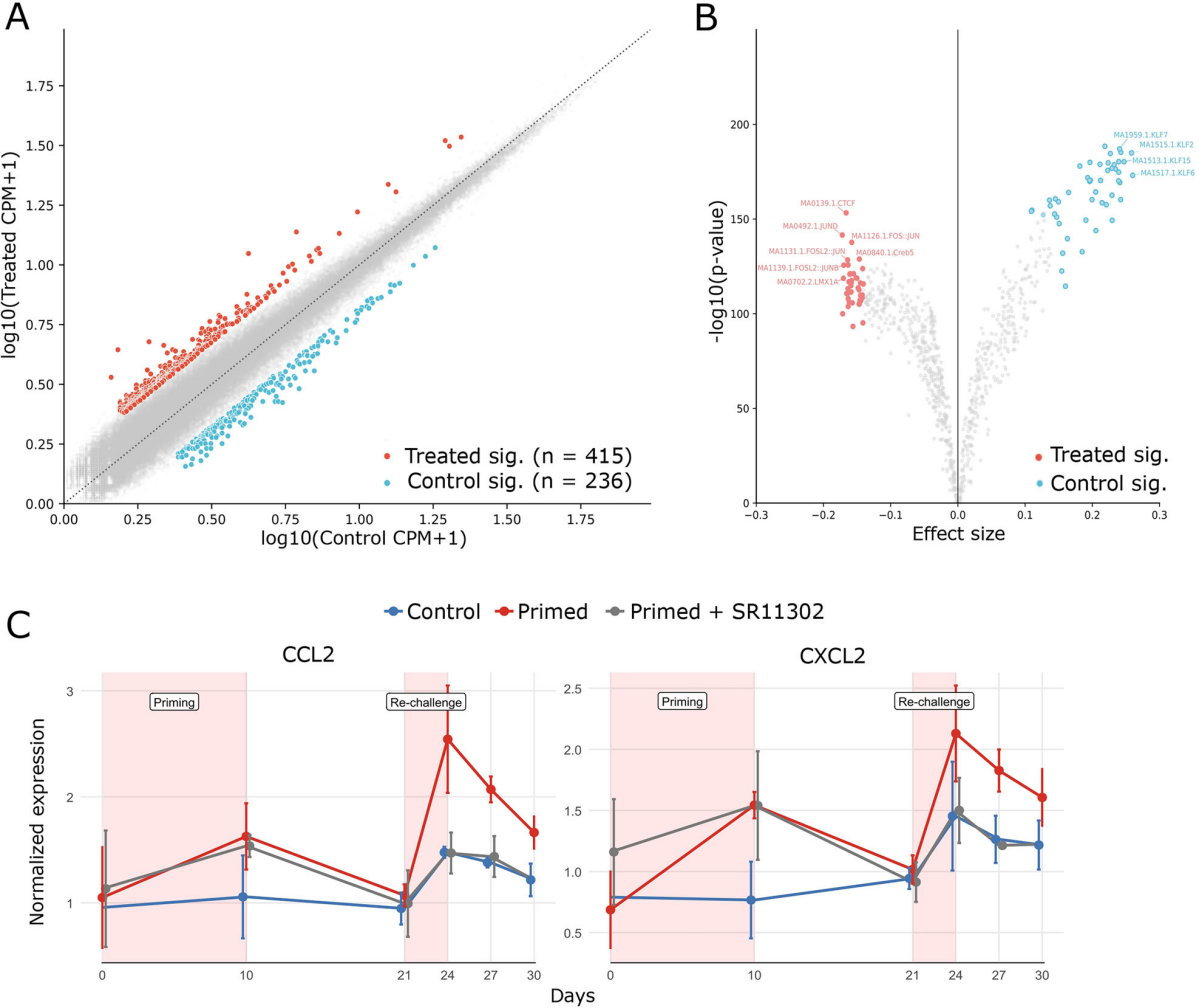
EGF + TGF-β exposure alters chromatin accessibility in primary human FAPs. **A** Two-sample ATAC-seq scatterplot. Each dot is a union peak (N = 139,260). Axes show log10(CPM + 1) for Control (x) and EGF + TGF-β (y). CPMs were derived from fragment counts per peak divided by library size, then log-transformed with a + 1 offset. Peaks were considered significant if the log2 fold change was ≥0.58. Three biological replicates were pooled per group. **B** Volcano plot indicating the transcription factor binding sites (TFBS) comparing EGF + TGF-β (treated) to vehicle (control). The x-axis is the effect size calculated as the change in the TFBS between groups. The y-axis is −log10(*p* value). The full list of results is in Suppl. File 1. **C** qPCR time-course for CCL2 and CXCL2. Mean ± SD of normalized expression over time for Control (blue), Primed (EGF + TGF-β; red), and Primed + SR11302 (gray). FAPs were exposed to EGF + TGF-β or vehicle at day 0 to 10 (priming) and 21 to 27 (re-challenge). *N* = 3 per group. CPM counts per million, EGF epidermal growth factor, TGF-β transforming growth factor beta.

We isolated primary human FAPs from non-diseased muscle biopsies and exposed them to TGF-β plus EGF, then profiled chromatin accessibility by ATAC-seq. Here, we identified 651 differentially accessible regions (DARs) between treated and control cells (Fig. 6A), indicating chromatin remodeling upon cytokine stimulation. To connect accessibility changes to TF activity, we analysed the footprinting profiles of both conditions and inferred differential binding of TFs [37]. Indeed, we detected a coordinated gain of AP-1 family activity in treated FAPs, including TF activity for JUN, JUND, JUNB, and related bZIP factors such as CREB5, while KLF family motifs had lower activities (Fig. 6B and Suppl. File 2). These patterns are consistent with AP-1’s known role as a stimulus-responsive regulator and with reports that several Krüppel-like factors (e.g., KLF15) inhibit pro-fibrotic programs in fibroblasts [38, 39].

Because AP-1 may establish or maintain accessible enhancer states, we next asked whether cytokine exposure induces a “primed” state in FAPs. Here, we define priming as a cellular state induced by prior stimulation that renders signaling pathways more responsive to a subsequent challenge, thereby amplifying the subsequent transcriptional response. We therefore performed a two-step experiment: FAPs were first treated with TGF-β and EGF (priming), rested in cytokine-free medium, and then re-challenged with the same cues (Fig. 6C). We measured the expression of *CCL2* and *CXCL2* – pro-inflammatory cytokines associated with an activated FAP phenotype [18, 40]. Both genes were predicted by our CCC model to be induced by the TGF-β/EGF axis. Upon re-challenge, primed FAPs mounted a stronger induction of *CCL2* and *CXCL2* than naïve cells, consistent with a primed state. To demonstrate the relevance of the AP-1 TF, we applied SR11302 during the re-challenge. SR11302 selectively blocks AP-1-dependent transactivation [41]. AP-1 inhibition blunted the induction of *CCL2* and *CXCL2*, indicating that AP-1 activity contributes to a primed phenotype and couples cytokine signaling to pro-inflammatory gene programs in FAPs.

## Discussion

IIMs are chronic, relapsing diseases in which therapeutic resistance remains a central challenge. Sustained remission is achieved in only 20 to 60% of patients, depending on cohort characteristics and clinical definitions [42–44]. This raises the question: Why does inflammation persist in muscle tissue despite immunosuppressive therapy? While the role of infiltrating immune cells is established in autoimmune diseases, a growing body of evidence implicates tissue-resident cells as active participants in inflammation. Notable examples include microglia, tissue-resident immune cells, in neuroinflammation [45, 46]. or synovial fibroblasts, tissue-resident non-immune cells, in rheumatoid arthritis [11, 12].

In skeletal muscle, the FAP lineage constitutes the main tissue-resident stromal population [4, 47]. In this study, we describe distinct, disease-specific FAP phenotypes that adapt to the prevailing immune context in their respective IIM subtype. For instance, FAPs in IBM favor T cell activation, while those in IMNM promote myeloid leukocyte activation. Across the IIM spectrum, a shared feature is the differentiation of the FAP lineage and adoption of a transcriptionally committed phenotype characterized by a pro-inflammatory and pro-fibrotic reprogramming, that potentially amplifies tissue inflammation. Notably, the proportion of pro-inflammatory FAPs correlated with the disease duration, suggesting that chronic exposure to inflammatory cues drives this maladaptive differentiation.

These findings align with the broader concept of “tissue priming”, established in other autoimmune diseases, wherein resident cells acquire a disease-promoting phenotype. Fibroblasts appear to provide a cellular niche from which specialized homeostatic and disease-associated fibroblast phenotypes emerge. Indeed, in chronic inflammatory and fibrotic disease, fibroblast lineages are heterogeneous and divide into functionally distinct states rather than acting as a single uniform population [48]. At the same time, recent cross-tissue single-cell analyses suggest that specific fibroblast subsets are conserved across tissues, at least in mice, such as an adventitial fibroblast phenotype defined by *Pi16* expression and a parenchymal fibroblast phenotype defined by *Col15a1* expression [49]. In response to disease, however, the fibroblast lineage may engage tissue-specific pathogenic programs. In rheumatoid arthritis, synovial fibroblasts develop enhanced proliferative capacity, produce pro-inflammatory mediators, and contribute to leukocyte recruitment and joint destruction [7, 8]. Synovial fibroblasts may be classified into functionally discrete subsets with fibroblast activation protein α (FAPα) marking a pathogenic fibroblast phenotype [50]. This phenotype can be further divided into *THY1*^+^ immune effector fibroblasts and FAPα^+^ *THY1*^-^ destructive fibroblasts mediating distinct mechanisms of joint damage [50]. Likewise, fibrotic lung tissue contains multiple fibroblast states, including disease-emergent fibroblasts localized to fibroblastic foci [51]. Together, fibroblast heterogeneity reflects a conserved program in which resident mesenchymal cells are shaped by local disease cues into functionally specialized, primed states that couple immune activation to extracellular-matrix remodeling.

Viewed in this context, our findings place IIM-associated FAPs within a broader pathogenic framework, while also suggesting that skeletal muscle adopts unique phenotypes. Biologically, FAPs are a muscle-resident mesenchymal stromal population with bipotent differentiation potential into a fibroblast-like phenotype or into adipocytes [47]. However, this functional classification does not translate into a stable transcriptomic taxonomy. Across human skeletal muscle studies, a number of partially overlapping FAP phenotypes have been described, based on diverging marker panels. These populations include, among others, *MME*^+^ FAPs with high adipogenic potential [4]. fibroblast-like cells expressing *THY1* abundant in aged muscle [13]. or *RUNX2*^+^ FAPs that promote muscle fibrosis [52]. These classifications support that the FAP lineage is heterogenous and adapts to muscle conditions, such as fibrosis or ageing, but also highlight the need for a unified transcriptomic nomenclature for human FAPs.

Mechanistically, our CCC analysis identified TGF-β and EGF as dominant upstream regulators of FAP reprogramming. TGF-β, primarily derived from immune cells, was associated with the induction of pro-fibrotic and senescence-related gene programs. This aligns with prior knowledge as TGF-β is a key activator of the fibroblast lineage [53]. EGF, predominantly secreted by type 2 myofibers, emerged as a complementary paracrine signal reinforcing FAP differentiation by engaging the AP-1 TF network. This dual-input model underscores a bidirectional axis of communication between immune and structural compartments, wherein myofibers actively contribute to muscle inflammation and fibrosis via paracrine signaling.

To place these findings in a pathophysiological context, we propose a model in which immune cell infiltration initiates muscle inflammation. These cells interact with tissue-resident cells, including myofibers as recently demonstrated [3, 10, 54]. and FAPs to establish an inflammatory niche. Chronic exposure to inflammatory signals, particularly TGF-β, drives the reprogramming of FAPs toward a pathogenic state. While immunosuppressive therapies may effectively attenuate the activity of circulating immune cells, they are unlikely to impact tissue-resident stromal cells, which lack conventional immune markers and may evade pharmacological targeting [55, 56]. Consequently, reprogrammed FAPs may persist within the tissue and maintain a permissive niche for recurring or smoldering autoimmunity. Indeed, FAPs have been previously shown to organize disease-specific cellular niches in muscle and are required for the survival of tissue-resident macrophages [20]. Intervening in these pathways, or reprogramming committed FAPs, may disrupt the self-reinforcing loop of inflammation and fibrosis. Following this line of thought, insights from previous research into tissue priming may be translated to skeletal muscle. In murine models of rheumatoid arthritis, tissue priming is mediated by bromodomain and extraterminal domain (BET) proteins. These proteins control the transcription of a range of immunoregulatory genes by binding to acetylated histones [57]. Inhibition of BET has been shown to reset stromal programming and improve outcomes when combined with immunosuppressants [55]. While these findings remain preclinical, they underscore the possibility to explore novel strategies in IIM. Based on our results, potential targets may include the TGF-β or AP-1 axis. Of the two, TGF-β inhibitors have entered clinical trials, and drug repurposing may be a novel avenue to target FAPs as tissue-resident cell types [58, 59].

An important limitation of our study is the absence of in vivo perturbation experiments to prove causality and to determine whether FAPs are required for disease persistence or severity. Resolving this question will require functional interrogation in model systems that allow subtype-appropriate testing of stromal cells. This advance is also needed to test whether targeting TGF-β or AP-1 improves outcomes in IIMs. At present, this remains a major challenge in IIMs, as available murine models reproduce only selected components of the human disease spectrum. This challenge is particularly evident in IBM. Xenograft-based approaches recapitulate degenerative and inflammatory features of IBM, but depend on engraftment of human tissue into immunodeficient mice and thus have intrinsic limitations as mechanistic in vivo systems [60]. Conversely, a recent model combining muscle-specific lymphotoxin-α/β expression with impaired autophagy represents an important step toward an IBM-like phenotype in mice, including treatment resistance and degenerative pathology, however, without capturing the T-cell pathology that characterizes human IBM [61]. We therefore consider the lack of direct in vivo perturbation an important limitation of the present work, while also noting that this reflects a broader constraint of the field. Future studies in refined, subtype-specific models will be required to determine whether FAPs are functionally necessary for disease maintenance and progression.

In summary, our study expands the paradigm of tissue priming to include skeletal muscle and positions FAPs as amplifiers of inflammation in IIMs. Addressing the contribution of tissue-resident cells may enhance the efficacy of existing treatments for IIM.

## Methods

### Ethics statement

The study was conducted in accordance with the Declaration of Helsinki and approved by the ethics committee of the Heinrich Heine University Dusseldorf (2016-053-f-S and 2021-1417) and the Charité-Universitätsmedizin Berlin (EA2/163/17). All patients signed written informed consent before the acquisition of the biopsy and clinical metadata.

### Patient recruitment and clinical data

Patients were recruited from two tertiary centers specialized in the management of IIM (University Hospital Duesseldorf and Charité- Universitätsmedizin Berlin). Patients were recruited from January 2014 to January 2024. Diagnosis was established based on histopathological assessments. IBM and IMNM patients were required to meet the European Neuromuscular Centre (ENMC) criteria for diagnosis [14, 62]. ASYS patients were required to meet the EULAR-ACR criteria for diagnosis [63]. Non-diseased controls (NDCs) served as the control cohort. As previously reported, these donors underwent muscle biopsy for diagnostic purposes, e.g., for myalgia [3]. They were required to have no objective muscle weakness or abnormal creatine kinase levels. For muscle histology, these biopsies were required to have no signs of inflammation or any other structural abnormalities. These patients had no myositis-specific or myositis-associated antibodies. This study included 22 patients. The individual number of patients is given for each experiment as indicated. The disease duration was defined as the time between the first symptoms as reported by the patients and the time of the muscle biopsy.

### Single nuclei isolation from muscle biopsies

All skeletal muscle specimens were cryopreserved at −80 °C before analysis according to the predefined standard operating procedure at the local biobank.

Single nuclei were isolated from cryopreserved muscle biopsy specimens. Approximately 60 mg of muscle was used for each sample. All biopsies were taken from the quadriceps muscle (vastus medialis) ~2 to 3 cm proximal to the knee joint.

The single nuclei suspension was processed as previously described [3]. using the GEXSCOPE^®^ Single Nucleus RNA Library Kit V2 (Singleron Biotechnologies). The tissue was immersed in a cold nucleus separation solution and dissociated. Further homogenization was achieved by performing five strokes with pestle A and five strokes with pestle B of the Kimble douncer (KIMBLE^®^ KONTES^®^ Dounce Tissue Grinder). The sample was then incubated on ice for 15 min, where the state of dissociation was monitored every 5 min under a light microscope. Following homogenization and digestion, the suspension was filtered using a 40-µm sterile strainer. The nuclei suspension was centrifuged at 200×*g* for 2 min at 4 °C, and the supernatant was centrifuged at 500×*g* for 5 min at 4 °C. The resulting pellet containing nuclei was resuspended in 0.25 ml cold nuclei suspension buffer. The quality of the nuclei was assessed by Trypan Blue staining (0.4% w/v, Gibco) under a light microscope. The nuclei were counted using propidium iodine with a Luna FX7 automated cell counter (Logos Biosystems, Villeneuve d’Ascq, France).

### Library generation and sequencing

Per sample, a total of 30,000 nuclei were loaded onto a microfluidic chip (Singleron GEXSCOPE^®^ Single Nucleus RNA Library Kit V2) for a minimal capture of 6000 nuclei. Barcoded beads containing a unique cell barcode were loaded into the chip, and nuclei were lysed. After nuclei lysis, polyadenylated RNA was captured onto the Barcode Beads by the poly (dT) sequence. Barcode Beads with captured mRNA molecules were collected and subjected to a reverse transcription reaction. The cDNA was then amplified and tested for quality control. NGS libraries generated were sequenced on an Illumina NovaSeq 6000 instrument using a paired-end 150 bp approach. The reads were demultiplexed on Illumina’s BaseCloud, and fastq files were used to initiate data analysis.

### Preprocessing of snRNA-seq data

Fastq files were processed to gene expression matrices using CeleScope™ (v1.14.1., www.github.com/singleron-RD/CeleScope; Singleron Biotechnologies). Briefly, fastq files were demultiplexed based on their cell barcodes and UMIs. Adapter sequences and poly-A tails were trimmed and aligned to the GRCh38 version of the human genome with Ensembl version 92 gene annotations (STAR v2.6.1a_08-27 and featureCounts 2.0.1). We excluded cells with a unique feature count over 2500 or below 200 or cells with a mitochondrial ratio of more than 5%. A combined expression matrix was constructed from all sequenced experiments, for downstream analysis.

### Clustering, differential expression, and enrichment analysis

snRNA-seq data were processed using the Seurat pipeline (version 5.2.1) [64]. Nuclei were filtered based on the quality metrics above. Data normalization and identification of highly variable genes were performed, followed by scaling and principal component analysis (PCA) for dimensionality reduction. To account for batch effects and integrate multiple datasets, Harmony integration was employed via the IntegrateLayers function. Clustering was conducted using the Seurat pipeline, and clusters were visualized using uniform manifold approximation and projection (UMAP) [64]. Cell types were annotated based on canonical marker genes.

Differential expression analysis between groups or conditions was performed using Seurat’s FindMarkers or FindAllMarkers functions. Subsequently, differentially expressed genes were subjected to functional enrichment analysis using the clusterProfiler package (version 4.16.0) [65]. Gene set enrichment analysis was conducted to identify enriched biological processes and pathways using the Gene Ontology (GO) Biological Pathways database.

### Trajectory analysis

To infer dynamic cellular trajectories and lineage relationships, we utilized the Palantir framework (version 1.4.0). The analysis was performed using the UMAP embedding obtained from the integrated Seurat object as the input manifold. We computed the pseudotime, entropy, and fate probabilities. Pseudotime was estimated by initializing the trajectory from a manually selected early-state population.

### Cell-to-cell communication analysis

Cell-to-cell communication analysis was performed using NicheNet (version 2.2.0) to predict ligand–target interactions between sender and receiver populations. For each disease group, we computed a NicheNet model using NDC as the reference. Following the standard NicheNet pipeline, differentially expressed genes in receiver cells were linked to potential upstream ligands expressed by sender cells. Ligand activity scores were computed based on their ability to predict the observed transcriptional response. The NichNet ligand–target prior model, ligand-receptor network, and weighted integrated networks were used as a database.

### Spatial RNA-seq

Sections from frozen muscle samples (10 µm) for eight patients (*n* = 2 per condition) were stained with hematoxylin and eosin (H&E) and processed following the manufacturer’s recommendations using the Visium Spatial Gene Expression kit (10x Genomics, Pleasanton, CA, USA). Visium libraries were sequenced using a NextSeq Illumina sequencer at a sequencing depth going from 40 to 100 M read-pairs per sample, depending on the section size.

Alignment and mapping were done using SpaceRanger v1.0.0 (10X Genomics) with GRCH38 v93 reference genome. The analysis pipeline used (v5.2.1) and semla packages (v1.4.0).

Cellular prediction derived from snRNAseq was used to infer cell type abundances in spatial transcriptomics spots using cell2location (v0.1.4). Raw counts from both datasets were used; negative binomial regression was performed to estimate cell type-specific expression profiles. The model was then trained using snRNAseq annotation and used to deconvolve the spatial data, estimating the expected abundance of each cell type per location using recommended parameters. The 5% posterior quantile was retained as a conservative measure of cell presence.

Spatial co-localization was assessed by computing neighbor-based cross-correlations using a spatial neighbor graph. Spatial projection of dominant cell types were produced using the MapMultipleFeatures function from semla.

### Isolation and culture of primary human FAPs

Muscle biopsies were dissociated utilizing a skeletal muscle dissociation kit and a gentleMACS™ Dissociator (#130-093-235, Miltenyi Biotec) according to the manufacturer’s instructions. From the resulting cell mixture, FAPs were isolated via magnetic-activated cell separation (MACS) by using PDGFRA as a marker (#MAB1263, R&D Systems, USA, Minneapolis) coupled with microbeads (Miltenyi Biotec). Purity was confirmed by staining for CD56, CD31 and PDGFRA. Only cells with a purity of >90% were used for further experiments. To cultivate the FAPs, the positive fractions of the MACS isolation were seeded in T75 flasks with Nunc Delta Surfaces (#156472, Thermo Fisher Scientific) and fibroblast growth medium (#C-23010, PromoCell) with 10% fetal calf serum (#A5209502, FCS; Thermo Fisher Scientific) and 1% penicillin/streptomycin (#15140122, Thermo Fisher Scientific).

After reaching a confluence of 70 to 80%, the medium was supplemented with 10 ng/µL EGF (#130-093-825, Miltenyi Biotec) and 5 ng/µL TGF-β1 (#130-095-066, Miltenyi Biotec). PBS was utilized as a control. Every 3 days, the medium was renewed according to the treatment (or control) group for a total duration of 10 days. After the treatment, FAPs were harvested for ATAC-seq analysis or for a treatment cycle. For the latter, FAPs were remained in TGF-β1/EGF-free medium for 11 days. Then, FAPs were treated again for three days and harvested, as indicated, for analysis by PCR using the same doses and schedule as above. A group of FAPs were additionally treated with the AP-1 inhibitor SR11302 at a concentration of 10 µM for the duration of the second exposure to TGF-β1/EGF. A total of three samples were pooled for each group and harvested for analysis by ATAC-seq.

### Assay for transposase-accessible chromatin using sequencing (ATAC-seq)

ATAC-seq library preparation and sequencing reactions were conducted at Azenta Life Sciences (South Plainfield, NJ, USA). Live cell samples were thawed, washed, and treated with DNAse I (Life Tech, Cat. #EN0521) to remove genomic DNA contamination. Live cell samples were quantified and assessed for viability using a Countess automated cell counter (Thermo Fisher Scientific). After cell lysis and cytosol removal, nuclei were treated with Tn5 enzyme (Illumina, Cat. #20034197) for 30 min at 37 °C and purified using the Minelute PCR Purification Kit (Qiagen, Cat. #28004) to produce tagmented DNA samples. Tagmented DNA was barcoded with Nextera Index Kit v2 (Illumina, Cat. #FC-131-2001) and amplified via PCR prior to a SPRI Bead cleanup to yield purified DNA libraries. The sequencing libraries were clustered on a flow cell. After clustering, the flowcell was loaded on an Illumina NovaseqX Plus according to the manufacturer’s instructions. The samples were sequenced using a 2 × 150bp paired-end (PE) configuration. Image analysis and base calling were conducted by the Control Software (CS). Raw sequence data (.bcl files) generated from the Illumina instrument were converted into fastq files and demultiplexed using Illumina’s bcl2fastq 2.17 software. One mismatch was allowed for index sequence identification.

### Alignment, peak calling, and differential analysis of ATAC-seq data

Paired-end FASTQ files were assessed with FastQC for per-base quality and adapter content. Reads were trimmed using fastp with adapter auto-detection and conservative quality trimming. Trimmed reads were aligned to the human reference genome (GRCh38/hg38) with BWA-MEM2 using read group tags for each sample. Alignments were processed with SAMtools [66]. Insert-size metrics were computed with Picard (CollectInsertSizeMetrics). To account for Tn5 insertion offsets and to standardize signal comparability, we ran deepTools alignmentSieve with --ATACshift, retained pairs with MAPQ ≥30, ignored marked duplicates, and excluded ENCODE blacklist regions (hg38). Mitochondrial reads (chrM/MT) were removed. Resulting BAMs were sorted and indexed. Coverage bigWig files were generated from filtered BAMs with deepTools bamCoverage using CPM normalization and 10-bp bins.

Peaks were called on filtered, duplicate-removed paired-end BAMs using MACS3 in paired-end mode with default *q*-value thresholding [67]. For downstream consensus, per-sample narrowPeak BEDs were combined into a union peak set. Library sizes were computed as the total number of kept fragments per sample after filtering. Peak counts were transformed to counts per million (CPM) using these library sizes. For each peak, we computed the mean CPM and the log2 fold change between conditions.

We inferred transcription factor (TF) occupancy from ATAC-seq using the TOBIAS workflow [37]. For each sample, we used the Tn5-shifted, mitochondrial-filtered BAM as input and the union of MACS3 peaks as regions. We ran [1]. ATACorrect to learn and remove Tn5 sequence bias and to generate corrected accessibility bigWigs; [2]. FootprintScores to compute base-resolution footprint scores within the union peak set; and [3]. BINDetect to scan motifs and test differential binding between conditions. BINDetect was run with the JASPAR 2022 vertebrate non-redundant motif set, the hg38 reference genome, and the union peaks. Unless stated, defaults were used. BINDetect reports an effect size (directional change in predicted TF binding) and a significance (−log₁₀ *p* value) after multiple-testing correction across motif instances.

### Quantitative RT–PCR

Primary human FAPs were cultured under the indicated conditions and sampled at 0, 10, 21, 24, 27, and 30 days. Total RNA was isolated and reverse-transcribed to cDNA as previously described [17]. qPCR was performed with SYBR Green on a real-time PCR system, following manufacturer instructions. Target genes and primers (OriGene) were: CCL2/MCP-1 (NM_002982): Forward 5′-AGAATCACCAGCAGCAAGTGTCC-3′, Reverse 5′-TCCTGAACCCACTTCTGCTTGG-3′ and CXCL2/GROβ (NM_002089): Forward 5′-GGCAGAAAGCTTGTCTCAACCC-3′, Reverse 5′-CTCCTTCAGGAACAGCCACCAA-3′. *ACTB* served as the housekeeping. For a given timepoint and group, the group’s day 0 mean ΔCt was used as the calibrator to compute ΔΔCt = ΔCt(sample) − meanΔCt (group day-0, same gene), and normalized as expression 2^−ΔΔCt^. Technical replicates were averaged at the 2^−ΔΔCt^ level; summary statistics are reported as mean ± SD per timepoint and group.

### Immunofluorescence

Fresh-frozen biopsies were cryosectioned at 8 µm. Sections were fixed in 4% paraformaldehyde (10 min), rinsed twice in PBS, washed 5 min in PBS, permeabilized in PBS with Triton X-100 (5 min), rinsed twice in PBS, then washed 5 min in PBS with Tween-20. After two PBS rinses, nonspecific binding was blocked with 5% normal goat serum (NGS) in PBS for 30 min. Primary antibodies were applied overnight at 4 °C in blocking buffer: rabbit anti-NOVA1 (1:500, Invitrogen, #PA5-95571. The next day, sections were rinsed twice in PBS and washed 5 min in PBS, followed by incubation with species-appropriate secondary antibodies for 1.5 h at RT (goat anti-mouse Cy3, 1:500, Jackson ImmunoResearch, #115-165-003; goat anti-rabbit Alexa Fluor 488, 1:500, Jackson ImmunoResearch, #111-545-003). A directly conjugated mouse anti-CD45 (CoraLite Plus 647, 1:20; Proteintech, #CL647-65082) was included during the secondary-antibody step. Nuclei were counterstained with DAPI (5 min), sections were rinsed (2× PBS, then 5 min PBS) and mounted in 70% glycerol in PBS. Images were acquired on a Keyence BZ-X1000 microscope (Keyence, Frankfurt). Acquisition settings (laser power, detector gain, and pinhole) were kept constant within each experiment.

### Statistical analysis

Statistical analysis was performed using *R* 4.4.3. Data were presented as median with IQR, mean with standard deviation (SD), as absolute (*n*) or relative frequencies (%). Differences between two groups were assessed using either Student’s *t*-test or the Mann–Whitney *U*-test, depending on data distribution. For comparisons involving more than two groups, one-way ANOVA or the Kruskal–Wallis test was applied, as appropriate. *p* ≥ 0.05 was classified as not significant, *p* < 0.05 as significant (*), *p* < 0.01 (**), *p* < 0.001 (***).

## Supplementary information

Suppl. Fig. 1

Suppl. Fig. 2

Suppl. Fig. 3

Suppl. Fig. 4

Suppl. File 1

Suppl. File 2

Suppl. File 3

## Data Availability

The complete snRNA-seq dataset is publicly available at https://tinyurl.com/FAPAtlas. Any other data will be provided upon request from a qualified investigator.
