# Supplementary material for: Inflammation reprograms fibro-adipogenic progenitors to sustain immunopathogenic niches in myositis
Source: Cell Death Dis. 2026 Jun 12;17(1):567. doi: 10.1038/s41419-026-08966-w (PMC13263347; doi:10.1038/s41419-026-08966-w)
Supplement: Supplementary file 1 — Suppl. Fig. 1 [file 41419_2026_8966_MOESM1_ESM.pdf]

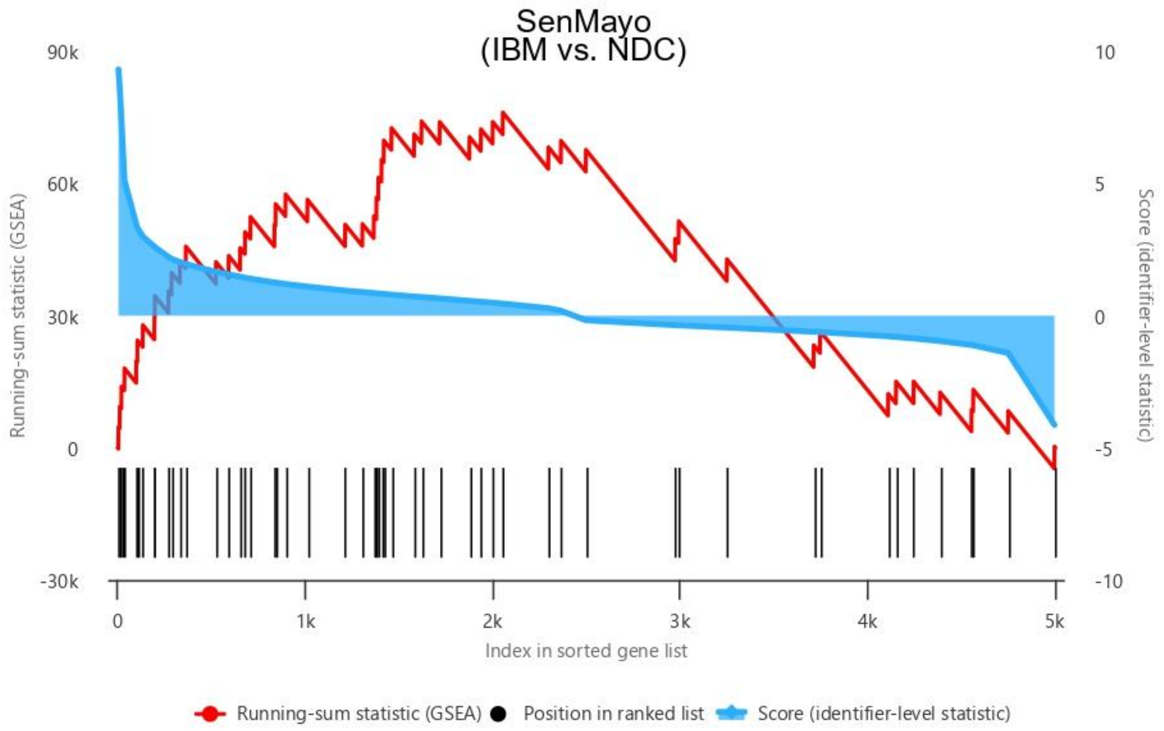

Suppl. Fig. 1: GSEA for the SenMayo dataset. DEGs were calculated for the FAP cell type for each IIM subtype compared to NDC. GSEA enrichment was performed on the list of significant DEGs. ASYS and IMNM are not shown as there was no significant enrichment for SenMayo.

Abbreviations: *ASYS, anti-synthetase syndrome; DEG, differentially expressed genes; FAP, fibro-adipogenic progenitor; GSEA, gene set enrichment analysis; IBM, inclusion body myositis; IMNM, immune-mediated necrotizing myopathy; NDC, non-diseased control.*
