# Supplementary material for: Inflammation reprograms fibro-adipogenic progenitors to sustain immunopathogenic niches in myositis
Source: Cell Death Dis. 2026 Jun 12;17(1):567. doi: 10.1038/s41419-026-08966-w (PMC13263347; doi:10.1038/s41419-026-08966-w)
Supplement: Supplementary file 2 — Suppl. Fig. 2 [file 41419_2026_8966_MOESM2_ESM.pdf]

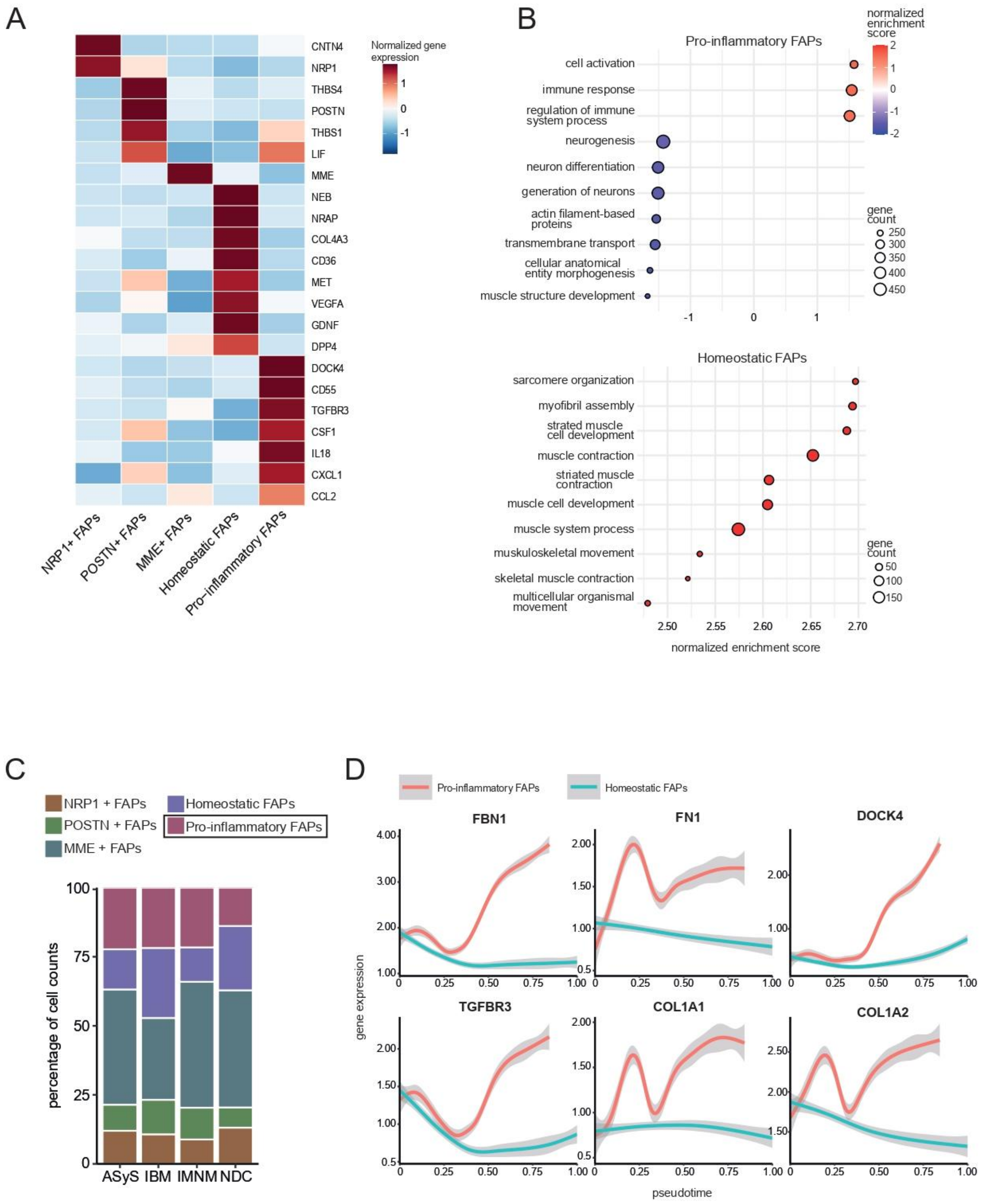

Suppl. Fig. 2: Phenotypes of FAP trajectories. **(A)** Heatmap of the top variable genes for the FAP population and its subclusters. **(B)** GSEA for the pro-inflammatory and homeostatic FAPs sorted by the NES. The top ten pathways are shown. For the pro-inflammatory FAPs, three pathways were upregulated and seven downregulated; for the homeostatic FAPs, all then pathways were upregulated. **(C)** Cell compositions for

the FAP subclusters across groups as stacked bar plots. **(D)** Gene expression trends across the pseudotime of the indicated FAP trajectories.

Abbreviations: *ASYS, anti-synthetase syndrome; DEG, differentially expressed genes; FAP, fibro-adipogenic progenitor; GSEA, gene set enrichment analysis; IBM, inclusion body myositis; IMNM, immune-mediated necrotizing myopathy; NDC, non-diseased control; NES, normalized enrichment score.*
