# Supplementary material for: Inflammation reprograms fibro-adipogenic progenitors to sustain immunopathogenic niches in myositis
Source: Cell Death Dis. 2026 Jun 12;17(1):567. doi: 10.1038/s41419-026-08966-w (PMC13263347; doi:10.1038/s41419-026-08966-w)
Supplement: Supplementary file 3 — Suppl. Fig. 3 [file 41419_2026_8966_MOESM3_ESM.pdf]

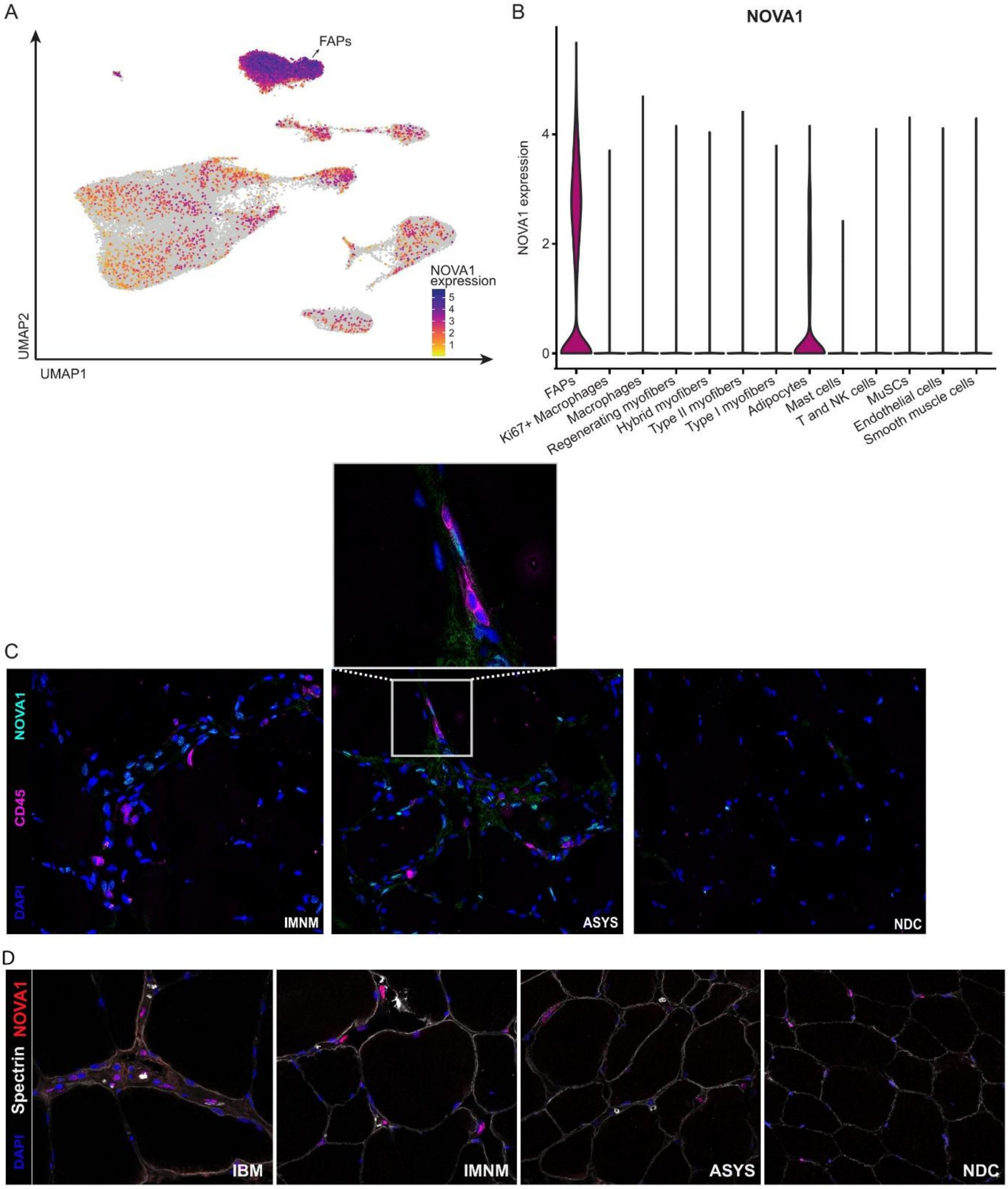

Suppl. Fig. 3: NOVA1 as a FAP marker. **(A)** Feature plot showing expression of *NOVA1* in the dataset. FAPs are indicated. **(B)** Violin plot indicating *NOVA1* expression across celltypes. *NOVA1* is expressed in FAPs and, to a lower degree, in their adipocyte differentiation stage. **(C-D)** Exemplary immunofluorescence images demonstrating CD45+ and NOVA1+ cells in IMNM, ASYS and in NDC.

Abbreviations: *ASYS, anti-synthetase syndrome; FAP, fibro-adipogenic progenitor; IMNM, immune-mediated necrotizing myopathy; NDC, non-diseased control; NOVA1, RNA-binding protein Nova-1.*
