# Supplementary material for: Inflammation reprograms fibro-adipogenic progenitors to sustain immunopathogenic niches in myositis
Source: Cell Death Dis. 2026 Jun 12;17(1):567. doi: 10.1038/s41419-026-08966-w (PMC13263347; doi:10.1038/s41419-026-08966-w)
Supplement: Supplementary file 4 — Suppl. Fig. 4 [file 41419_2026_8966_MOESM4_ESM.pdf]

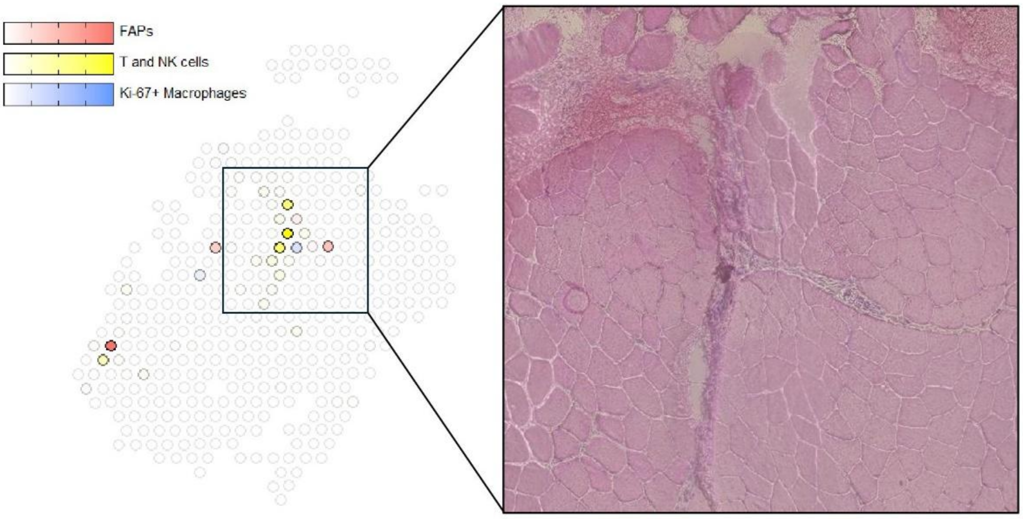

Suppl. Fig. 4. Enrichment for FAPs, T and NK cells, and Ki-67+ macrophages in an exemplary NDC tissue sample. Spots enriched for immune cells correlate with blood vessels on the H&E staining. Only a small number of FAPs were detected in the parenchyma.

Abbreviations: *FAP, fibro-adipogenic progenitor; NDC, non-diseased control.*
