# Supplementary figures and images for: Inflammation reprograms fibro-adipogenic progenitors to sustain immunopathogenic niches in myositis
Source: Cell Death Dis. 2026 Jun 12;17(1):567. doi: 10.1038/s41419-026-08966-w (PMC13263347; doi:10.1038/s41419-026-08966-w)

A

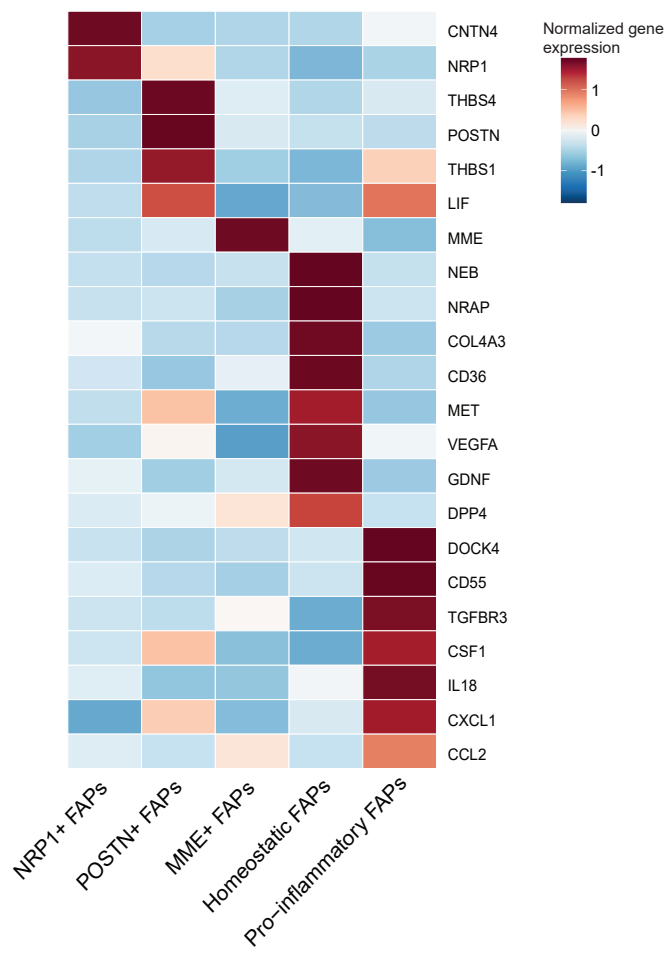

B

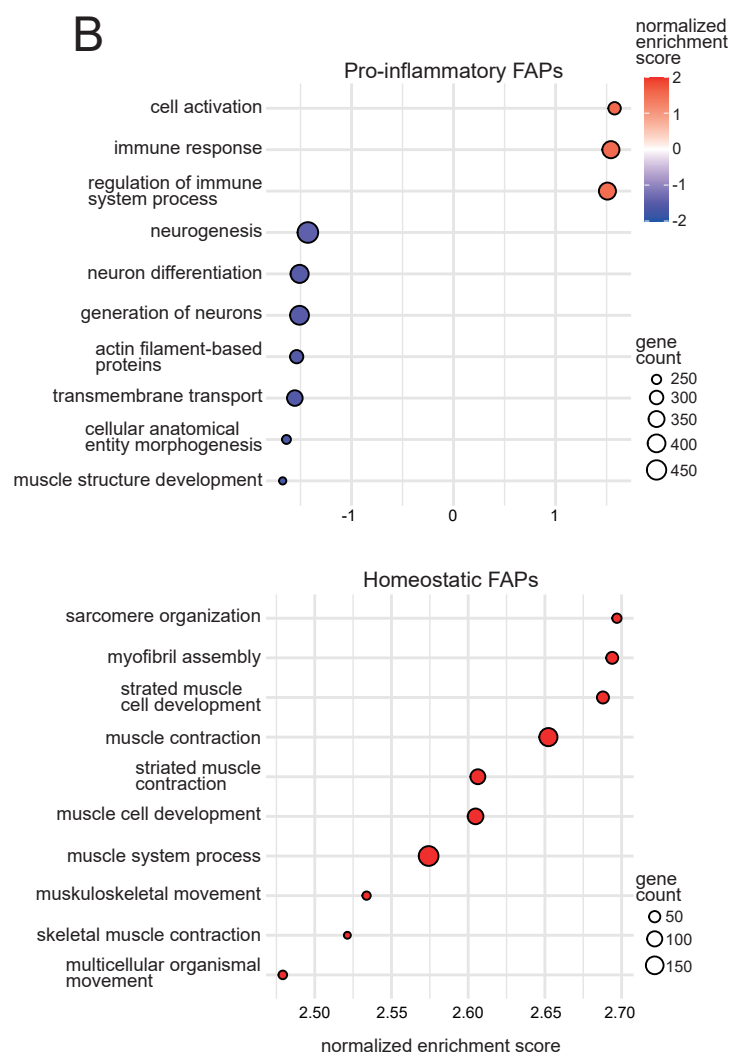

C

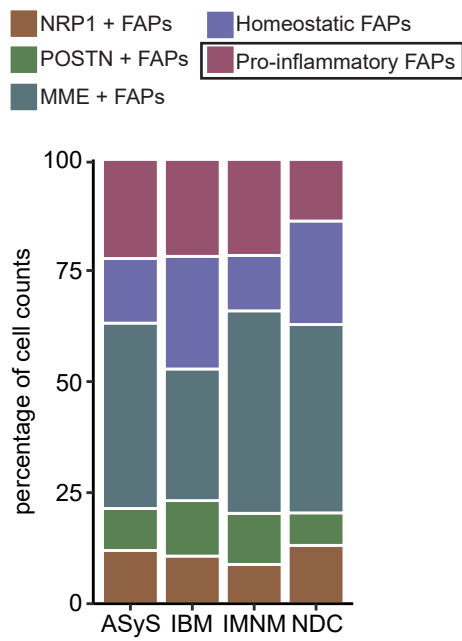

D

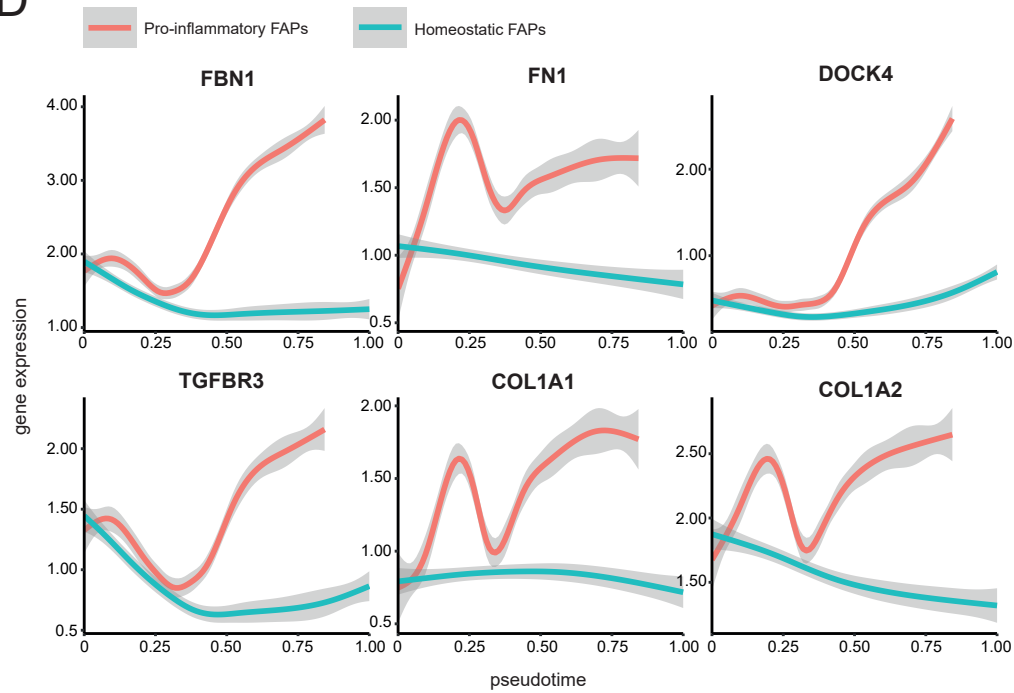

Supplement: Supplementary file 6 — Suppl. File 2 [file 41419_2026_8966_MOESM6_ESM.pdf]

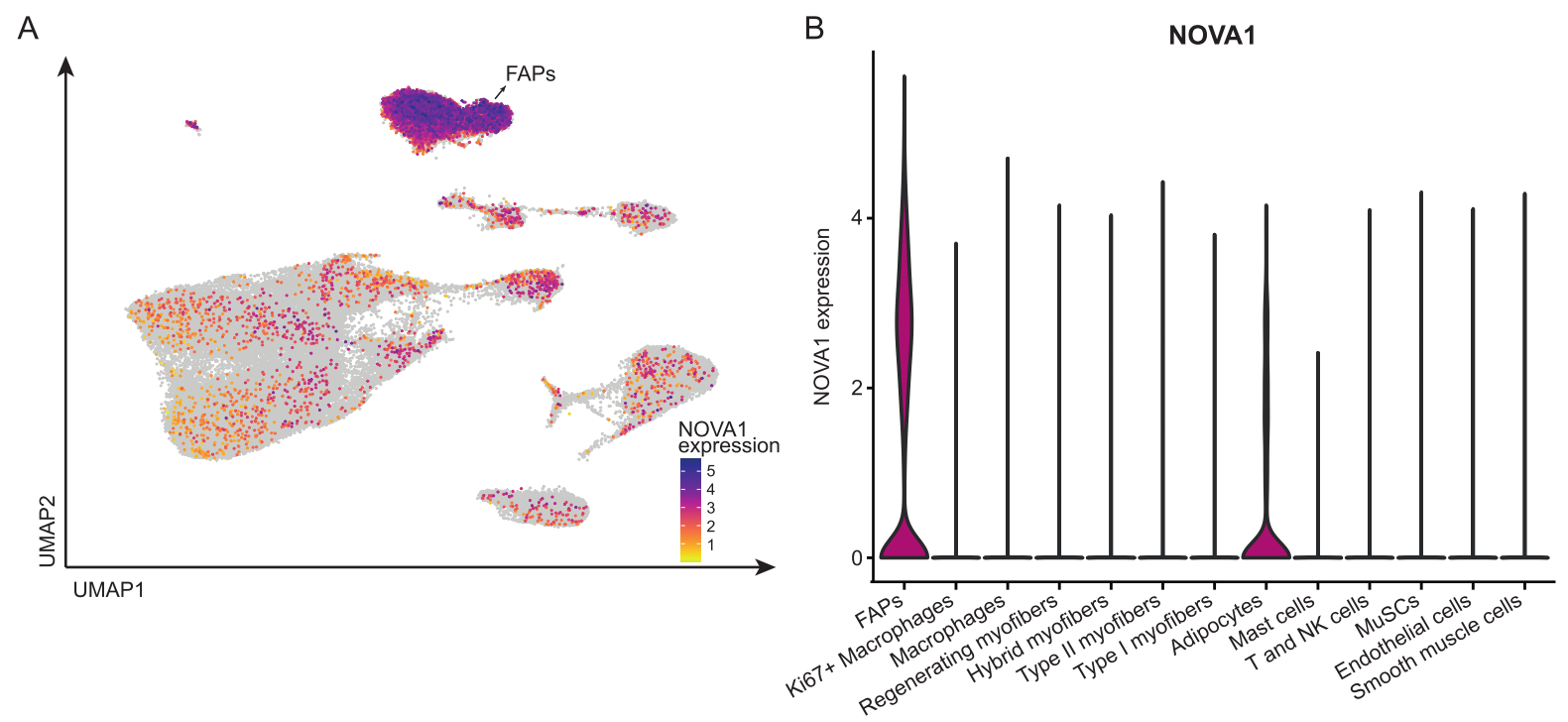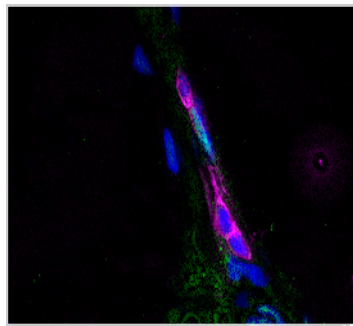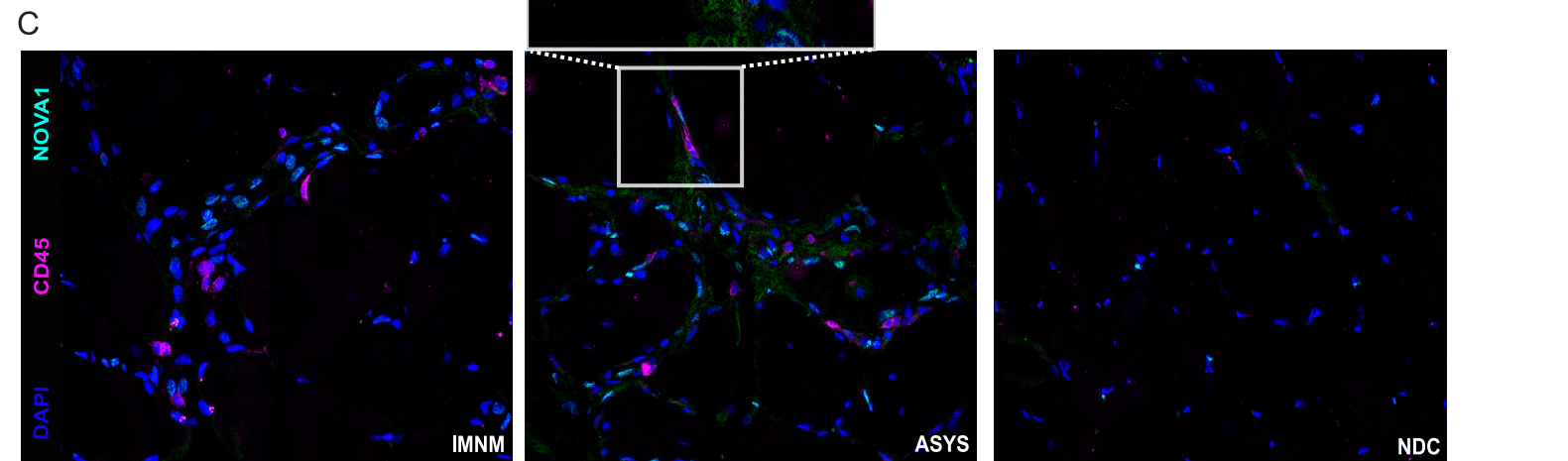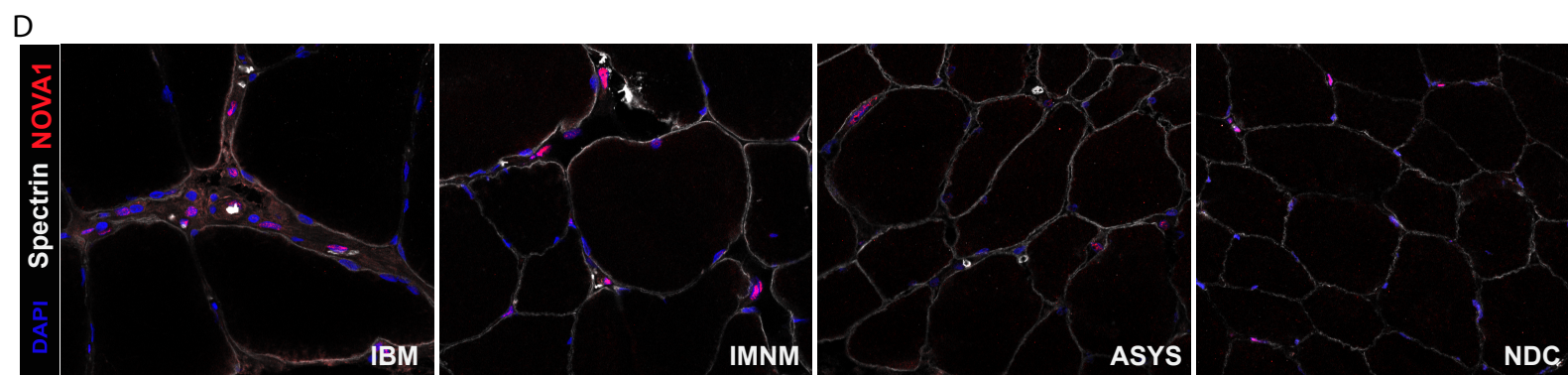

Supplement: Supplementary file 7 — Suppl. File 3 [file 41419_2026_8966_MOESM7_ESM.pdf]
